# miRNA-associated gene networks reveal potential candidate markers for Alzheimer’s disease

**DOI:** 10.3389/fmolb.2025.1699404

**Published:** 2026-03-06

**Authors:** Shujuan Pan, Yanfang Zhou, Xiaoyu Wang, Jinghui Tong, Yanli Li, Junchao Huang, Song Chen, Yimin Cui, Zhiren Wang, Yun-Long Tan

**Affiliations:** 1 Peking University HuiLongGuan Clinical Medical School, Beijing Huilongguan Hospital, Beijing, China; 2 Department of Pharmacy, Peking University First Hospital, Beijing, China

**Keywords:** Alzheimer’s disease, GAD1, gene networks, miRNA, SLC32A1

## Abstract

**Introduction:**

Alzheimer’s disease (AD) is a progressive neurodegenerative disorder characterized by memory and cognitive decline. Recent studies highlight the significant role of microRNAs (miRNAs) in regulating genes related to AD. This research aims to develop miRNA-associated gene regulatory networks as candidate AD biomarkers.

**Methods:**

We recruited 85 AD patients and 74 healthy controls, conducting whole blood miRNA sequencing and applying machine learning to identify differentially expressed miRNAs, which were validated by quantitative reverse transcription polymerase chain reaction (qRT-PCR). We used bioinformatics databases to predict target genes for these miRNAs and obtained gene expression data from the Gene Expression Omnibus (GEO) database (GSE122063 and GSE18309). Using the ggplot2 package in R, we discovered the overlap between miRNA target genes and differentially expressed genes (DEGs) from the GSE datasets. Encyclopedia of Genes and Genomes (KEGG) and Gene Ontology (GO) enrichment analyses of the DEGs were then conducted using the Metascape database. Key hub genes were pinpointed by constructing a protein–protein interaction (PPI) network with the Retrieval of Interacting Genes (STRING) database and analyzing it with cytoHubba. Drug-gene interactions were predicted and examined using the Drug–Gene Interaction database (DGIdb) (http://www.dgidb.org/).

**Results:**

qRT-PCR was used to confirm the expression of the hub genes. The results showed that four miRNAs (miR-192-5p, miR-484, miR-21-5p, and miR-24-2-5p) were downregulated, while two target RNAs (SLC32A1 and GAD1) were upregulated.

**Discussion:**

This regulatory network, which is strongly linked to AD, has been initially identified as a candidate biomarker for AD. Our research provides new insights into the pathogenic mechanisms of AD, potentially improving the understanding of miRNAs’ role in the disease.

## Introduction

Alzheimer’s disease (AD) is the predominant neurodegenerative disorder, characterized by cognitive and memory dysfunction. The disease progresses gradually and is more prevalent in older individuals. Pathologically, AD is characterized by the presence of senile plaques composed of abnormal amyloid-protein deposits outside neurons, as well as neurofibrillary tangles (NFTs) resulting from abnormal tau-protein phosphorylation (Johnson et al., 2020). Presently, AD and related dementias impact over 50 million individuals globally, with projections indicating a 68% increase in affected individuals by 2050 (Chen et al., 2024). Research on pharmacology has not yet produced effective treatments for AD, only symptom relief (Tatulian, 2022; Long and Holtzman, 2019; Wu and Fuh, 2025). A better understanding of the molecular mechanisms of AD could lead to new candidate and therapeutic options for patients.

In recent years, advances in bioinformatics tools and high-throughput sequencing technologies have allowed a better understanding of the occurrence and progression of diseases like AD (Xu et al., 2022). These technologies can be used for prediction screening, early diagnosis, prognosis, and personalized treatment. Differentially expressed genes (DEGs) and miRNAs can offer important insights for predicting the survival of AD patients. However, various factors, including sample heterogeneity, varied screening methods, diverse data mining techniques, and limited sample size, can lead to inaccurate findings in individual studies. To address this, integrated analysis utilizing combined datasets has emerged as a promising approach. Consequently, numerous recent studies have effectively utilized publicly available datasets, such as GEO, to discover novel candidate and prognostic molecular markers for the treatment of AD (Ou et al., 2021; Huang et al., 2024; Su et al., 2023; Gu et al., 2024).

To achieve this objective, aberrantly expressed miRNAs were initially identified through peripheral blood RNA sequencing of 35 samples from individuals with AD and 35 samples from healthy individuals, and their respective target genes were determined. Subsequently, an examination of key DEGS was conducted through gene expression profiling of the frontal and temporal cortex as well as peripheral blood mononuclear cells (PBMCs) in AD, utilizing bioinformatics analysis of the GEO database. The intersection of the identified target genes and DEGs was then determined, followed by an analysis of GO functional annotations and KEGG pathways. The PPI network was generated utilizing the STRING database. Hub genes were then identified within this network. Subsequently, key hub genes were validated through qRT-PCR, and their correlation with clinical symptoms was assessed using Pearson’s correlation coefficient. Differentially expressed key hub genes were further analyzed using the DGIdb2.0 to predict and construct a drug–gene network diagram.

Collectively, comprehension of the fundamental mechanisms driving the pathogenesis of AD at the molecular level has the potential to enhance the creation of innovative early diagnostic and therapeutic interventions, thereby enhancing the strategies and prognosis for individuals affected by AD. Our research elucidates the molecular mechanisms underlying the development and progression of AD and identifies key genes associated with early diagnosis and unfavorable outcomes of the disease.

## Methods

### Participants

Initially, 85 patients diagnosed with AD were selected from Beijing Hui Long Guan Hospital for inclusion in this study. AD diagnosis was confirmed based on criteria outlined in the *Diagnostic and Statistical Manual of Mental Disorders*, Fourth Edition (DSM-IV), with patients exhibiting a Mini-Mental State Examination (MMSE) score of less than 16. The MMSE, consisting of 20 items assessing immediate memory, general knowledge, recall, and orientation, was utilized to evaluate the severity of AD, with lower scores indicating greater cognitive impairment. The severity of AD is categorized based on MMSE scores, with mild cognitive impairment defined as MMSE scores ≥21 points, moderate cognitive impairment as scores ranging from 10–20, and severe cognitive impairment as scores ≤9 points.

The exclusion criteria for the study included a diagnosis of dementia other than AD, a history of schizophrenia or pure and obvious depressive symptoms, an acute or difficult-to-control physical illness, chronic alcoholism or drug dependence that may cause dementia, and the use of medications that may affect neurocognitive function. Healthy control participants (n = 74) were selected to match the age and gender of the AD group and had no history of mental illness or neurological disease in their family.

All participants provided written informed consent. The study was approved by the Institutional Ethical Committee of Beijing Huilongguan Hospital. All methods were performed in accordance with the appropriate guidelines and regulations.

### miRNA sequencing and analysis of differentially expressed miRNAs

A cohort comprising 35 individuals diagnosed with AD and 35 healthy controls was assembled for the study. Fasting venous blood samples of 10 mL were obtained from each participant, from which miRNAs were isolated using the miRNeasy kit. Subsequently, the QIAseq miRNA Library Kit was utilized to construct miRNA sequencing libraries which were then subjected to sequencing on an Illumina platform. Quality control of the sequencing data was performed using FastQC software, followed by an alignment of reads to the reference genome using Bowtie software. Differential expression analysis was conducted using edgeR. Low-quality reads were filtered out, and cleaned reads were aligned to the human genome with Hisat2. miRNA read counts were quantified using feature counts and normalized with the trimmed mean of M-values (TMM) method to obtain counts per million (CPM) values. edgeR’s generalized linear model (GLM) and likelihood ratio test (LRT) identified differentially expressed miRNAs, with significance set at a false discovery rate (FDR) < 0.05 and |log_2_FC| ≥1. For the differentially expressed miRNAs, the dataset was partitioned into a training set and a test set in a 7:3 ratio. A random forest (RF) algorithm was utilized for the analysis, and a fivefold cross-validation was conducted to assess model stability. miRNAs ranking in the top 20% based on variable importance scores were selected to focus on biologically relevant candidate molecules. During each fold of the cross-validation, weight values were assigned to these miRNAs to identify molecules with significant discriminative power between AD patient samples and healthy control samples. Specifically, for each differentially expressed miRNA, weights were calculated using LASSO regression, with the absolute values of the regression coefficients (|β|) serving as weight indicators. A larger |β| value signified a more substantial impact of the miRNA on disease classification or phenotype prediction. The final weights were determined by averaging the results from the fivefold cross-validation to mitigate the influence of random fluctuations, enhance the statistical validity of evaluation metrics, and ensure the robustness and reproducibility of the findings. Furthermore, the individual impact of each miRNA on the area under the curve (AUC) was assessed using receiver operating characteristic (ROC) analysis. This analysis facilitated the identification of miRNA combinations with significant classification efficacy. These candidate miRNA combinations, along with covariates such as age and sex, were employed as feature variables in the modeling process. Three models—logistic regression (LR), RF, and support vector machine (SVM)—were utilized for training sample classification. The classifiers for LR, RF, and SVM were configured with the parameter class_weight = balanced. This configuration allowed the algorithm to automatically adjust the loss function, thereby increasing the “cost” of misclassifying the minority class and ensuring greater attention to its accurate classification. Assessment metrics comprising ROC curves, accuracy, sensitivity, and specificity were employed to evaluate and compare the classification performance of the models utilizing candidate miRNA combinations. The evaluation of candidate miRNAs was predicated on three principal indicators: importance, expression level, and correlation. A LR model was employed to construct a miRNA candidate panel model, with coefficients, equations, and probabilities being computed accordingly. ROC analysis was conducted to assess the classification efficacy of the candidate miRNAs. An AUC value exceeding 0.9 is indicative of robust performance on the test dataset.

The analysis of differentially expressed miRNAs was performed using RT-qPCR. Total RNA was extracted from whole blood utilizing the miRNeasy Mini Kit (Qiagen, Germany), and its concentration and purity were assessed with a NanoDrop 2000 spectrophotometer, ensuring an A260/A280 ratio between 1.8 and 2.0. RNA integrity was confirmed through agarose gel electrophoresis. Subsequently, all miRNA targets were quantified via a polyadenylation-mediated qRT-PCR system provided by TIANGEN Biotech in Beijing, China, using poly(A) tailing for universal cDNA synthesis to replace stem-loop RT primers. The system integrated three core kits: an external reference spike-in control using the TIANGEN miRNA External Reference Kit CR100-01 added pre-extraction at 10^8^ copies/μL to monitor efficiency, a reverse transcription step with the TIANGEN miRcute Plus First-Strand cDNA Synthesis Kit KR211 that adds 15–20 A poly(A) tails via Poly(A) Polymerase and synthesizes cDNA with a universal oligo (dT) primer under 37 °C for 60 min for tailing and reverse transcription followed by 85 °C for 5 min for inactivation, and a qPCR step using the TIANGEN miRcute Plus qPCR Kit SYBR Green FP411 with SYBR Green I Master Mix, miRNA-specific forward primers, and a universal reverse primer targeting the poly(A) tail. Standard curves were generated with synthetic mimics in tenfold dilutions, showing R^2^ values greater than 0.99 and amplification efficiencies of 90%–110%. Negative controls included no-template controls and no-reverse-transcription controls. Triplicate runs were performed with a coefficient of variation below 5%, and normalization was done to spike-in using the formula ΔCt = Ct_target − Ct_spike-in and to U6 snRNA.

### Prediction of differentially expressed miRNA target genes

We utilized the miRWalk 2.0 tool to predict target genes by inputting differential miRNAs and integrating data from five databases (miRWalk, Microt4, miRanda, miRMap, and TargetScan). Subsequently, we obtained a list of predicted miRNA–target regulation pairs and filtered out those pairs present in all five databases to establish a comprehensive miRNA regulation network.

### Microarray data

The microarray data from GSE122063 and GSE18309 were retrieved from the GEO database (http://www.ncbi.nih.gov/geo). The GSE122063 dataset included 44 samples from individuals with healthy controls and 56 patients with AD, with gene expression profiling conducted on frontal and temporal cortex samples from AD cases and controls sourced from the University of Michigan Brain Bank. The GSE18309 dataset, comprising three samples from individuals with healthy controls and three with AD, was sourced from transcriptomes of PBMC and subjected to microarray analysis. The raw data were obtained in MINiML file format. Differential expression analysis of mRNA was conducted using the limma package in R (version 3.54.0). Linear models were fitted, and raw p-values were calculated through the application of empirical Bayes moderation. To account for multiple testing, the Benjamini–Hochberg (BH) procedure was employed to adjust the raw p-values, thereby controlling FDR. The threshold for statistical significance was established at an FDR of less than 0.05, with an additional criterion of an absolute log_2_ fold change (|log_2_FC|) of 1.

### Integrated analysis of miRNA target gene predictions and microarray expression data

An integrated analysis was performed on genomic data, incorporating predictions of differentially expressed miRNA target genes alongside microarray data. This approach involved the amalgamation of predicted target genes with DEGs identified through transcriptome sequencing. The intersection of these two gene sets was determined using Venn calculation software. Subsequently, the integrated data underwent GO and KEGG pathway enrichment analysis through the Metascape online website (http://metascape.org). This analysis aimed to identify significantly enriched biological functions and pathways within the integrated gene set.

### Construction of the predicted PPI network

The STRING database, a comprehensive online resource of known and predicted PPI, encompasses both direct (physical) and indirect (functional) associations. In this study, PPI analysis of DEGS was conducted using the STRING database (version 11.0), with a threshold combined score of greater than 0.9 deemed as statistically significant. Subsequently, visualization of the PPI network was achieved through Cytoscape (version 3.8.2). Lastly, hub genes within the DEGs were identified using cytoHubba (a Cytoscape plug-in) and the maximal clique centrality (MCC) method.

### Validation of hub gene expression

To validate the potential role of the hub genes, 5 mL of EDTA-anticoagulated whole blood was collected at 7:00 and 9:00 a.m. following an overnight fast. The RNA from the whole blood was immediately stored at −80 °C until analysis. Erythrocytes were lysed using the appropriate lysis buffer, and total RNA was extracted using the silica-membrane column purification method in strict accordance with the manufacturer’s protocol (Qiagen PAXgene Blood RNA Kit). The concentration and purity of the extracted RNA were measured using a NanoDrop spectrophotometer, ensuring an A260/A280 ratio between 1.8 and 2.0. An A260/A230 ratio greater than 1.8. RNA integrity was subsequently assessed using an Agilent bioanalyzer 2100 or an equivalent capillary electrophoresis system, requiring an RNA integrity number (RIN) of ≥7.0 and a normal 28S/18S rRNA ratio. Samples with a RIN of less than 7 or showing signs of degradation were excluded from further analysis. Whole-blood RNA brain-derived neurotrophic factor (BDNF), solute carrier family 32 member 1 (SLC32A1), glutamate decarboxylase 1 (GAD1), glutamate decarboxylase 2 (GAD2), synaptoporin (SYNPR), synaptosomal-associated protein 25 (SNAP25), calbindin 1 (CALB1), gamma-aminobutyric acid type A receptor alpha 1 subunit (GABRA1), gamma-aminobutyric acid type A receptor alphaBeta 2 subunit (GABRB2), and growth-associated protein 43 (GAP43) concentrations were determined by qRT-PCR. Reverse transcription of mRNA was performed using a high-capacity RNA-to-cDNA Kit (Invitrogen, United States) following the manufacturer’s instructions. qRT-PCR was conducted using a TaqMan® probe-based assay (Thermo Fisher Scientific, United States). PCR was performed with the 7900HT sequence detection system as follows: 95 °C for 2 min, 40 cycles of 95 °C for 15 s, and 60 °C for 60 s. Dissociation curves were plotted for every reaction to test the specificity of the amplification. Each qPCR assay included three technical replicates per sample, conducted as independent reactions using the same cDNA template. The mean Ct values were utilized for subsequent analyses. Samples with abnormal melting curves, or amplification efficiencies outside the acceptable range (<90% or >110%), were systematically excluded from the analysis. Furthermore, samples with GAPDH Ct values greater than 35, which suggest RNA degradation, were also excluded. Relative gene expression was calculated with the 2^−ΔΔCT^ method and normalized against the glyceraldehyde 3-phosphate dehydrogenase (*GAPDH*) gene. PCR primers were as Supplementary Table S1. The relationship between the expression level of hub genes and clinical symptoms was investigated.

### Prediction and analysis of small molecules of drugs

We utilized DGIdb2.0 (http://www.dgidb.org/) to identify genes associated with diseases. The drug database parameters were restricted to DrugBank and FDA-approved drugs. All drug–gene interaction pairs were predicted to construct a drug–gene network diagram. DGIdb facilitated the exploration of existing resources and generated hypotheses regarding gene targeting in therapeutic interventions or preferential use in drug development (Freshour et al., 2021; Cotto et al., 2018). Gene–drug pairs were prioritized based on the following criteria: (1) type of interaction between drug and gene, with “direct inhibition” or “indirect regulation” prioritized over “predicted associations”; (2) clinical stage of the drug, with phase III clinical trials prioritized over phases II, I, and approved but off-label indications; (3) the established relevance of the gene to AD pathology, with priority given to genes associated with amyloid-beta deposition or tau-protein abnormalities; (4) the confidence score in DGIdb, with only associations scoring at or above the median value included.

### Statistical analysis

Demographic and clinical data were analyzed using analysis of variance (ANOVA) for continuous variables and chi-squared test for categorical variables. A Mann Whitney U test or independent sample t-test was used to examine differences in miRNA, mRNA levels, and MMSE scores between groups. The distributions of hub-gene mRNA were skewed, and they were log_10_ transformed to a normal distribution prior to correlation analyses. Partial correlations were performed with age, sex, and education as covariates during the examination of the relationship between biological and neuropsychological variables. Data are presented as mean and standard deviation (mean ± SD). Statistical tests were considered significant at *p* < 0.05; all measures were centered, and age and sex were included in regression analyses. All statistical tests were two-tailed.

## Result

### Patients’ characteristics

The mean age of the healthy control group was 73.24 ± 9.29 years, while that of the AD patients was 75.79 ± 16.03 years. Statistical analysis revealed no significant differences in gender distribution (P = 0.07) or age (P = 0.24) between the healthy controls and AD patients. However, the MMSE scores were significantly higher in the healthy controls than in the AD patients (P < 0.001). Disease duration was calculated as the interval from symptom onset to study enrollment (mean ± SD: 0.74 ± 0.38 years) (Table 1).

**TABLE 1:** Demographic characteristics and mRNA expression levels of AD patients and healthy controls.

Demographics	AD (n = 85)	Controls (n = 74)	t or *X* ^2^	p	q
Sex (M/F)	33/52	39/35	3.37	0.07	0.175
Age (years)	75.79 ± 16.03	73.24 ± 9.29	1.18	0.24	0.369
MMSE	6.16 ± 5.91	29.17 ± 0.93	−32.18	<0.001**	0.005
Illness Duration (month)	8.83 ± 4.58	N/A	N/A	N/A	N/A

**p ≤ 0.001.

### Identification of differentially expressed miRNAs

**FIGURE 1:**
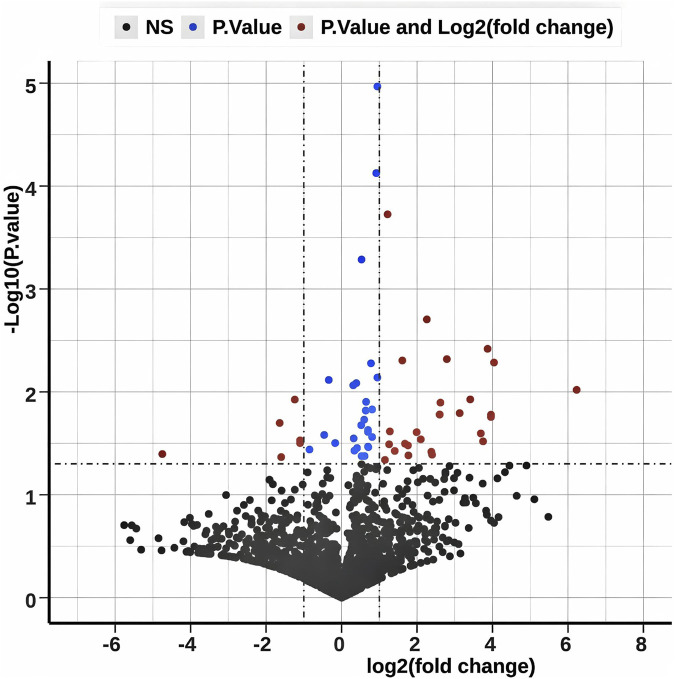
Differentially expressed miRNA information. The volcano plot illustrates the differential expression of miRNAs between AD patients and control subjects. White points indicate no significant difference in expression. Blue points denote miRNAs that are significant based solely on the P-value (p < 0.05 and ∣log2(FC)∣<1). Red points represent miRNAs that are significant based on both the P-value (p < 0.05) and the expression fold change (∣log2(FC)∣>1).

**FIGURE 2:**
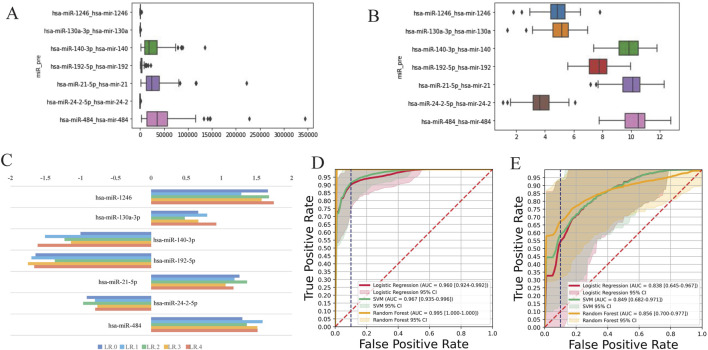
Bar plot of multivariable model coefficients for miRNAs. **(A)** Raw data show hsa-miR expression distribution, highlighting narrower ranges for genomic positions 56, 127, 445, and 459 compared to other markers, indicating data imbalances. **(B)** Standardized hsa-miR expression levels eliminate technical variations, allowing clearer biological comparisons and addressing distribution heterogeneity from Panel A. **(C)** Model performance is assessed through fivefold cross-validation of a LR model, with LR0–LR4 indicating performance on each fold. The average score across folds underscores the importance of cross-validation for robust conclusions. **(D)** Training set ROC curve, comparing the classification performance of RF, LR, and SVM, with AUC values indicating excellent model fit (RF: 0.995, LR: 0.96, SVM: 0.967). **(E)** Test Set ROC Curve, validating these algorithms on an independent set, with AUC values (0.838–0.856) confirming robustness (RF: 0.856, LR: 0.838, SVM: 0.849).

**FIGURE 3:**
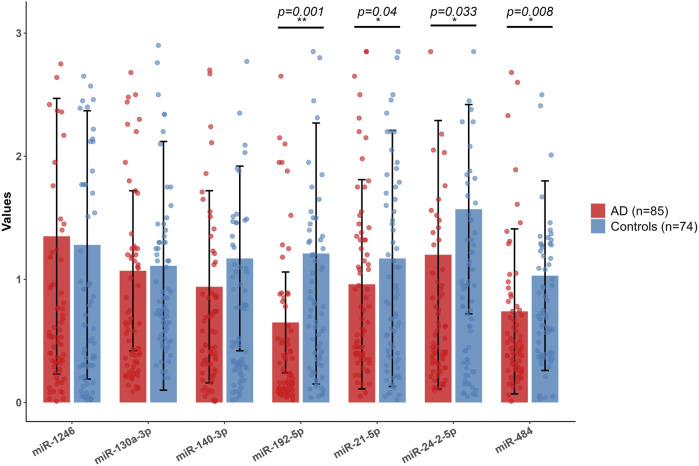
miRNA expression levels of AD patients and healthy controls by qRT-PCR. Red bars represent AD group (n = 85), and blue bars represent control group (n = 74). Black horizontal lines connect pairwise comparisons and indicate statistical tests. The p-values annotated above (p < 0.001, p < 0.05) denote the significance level of the differences.

Table 2 presents the demographics of 35 AD patients and 35 healthy controls in the miRNA sequencing cohort. The AD group had a male/female ratio of 12/23, while the control group had 21/14, with a χ^2^ test result of 4.64 and a p-value of 0.05 (q = 0.075). The average age was 76.17 ± 7.68 years for the AD group and 75.34 ± 10.05 years for the control group, showing no significant difference (t = 0.39, p = 0.70, q = 0.7). The AD group had a significantly lower MMSE score (5.83 ± 5.42) than controls (36.74 ± 2.08) with t = −27.13, p < 0.001, q = 0.003. Illness duration for the AD group was 8.94 ± 4.84 months. The inclusion criteria for miRNAs selected after sequencing were defined through a three-step progressive screening process: initial filtering of low-quality signals and low-expression samples with strict quality control, retaining 2065 qualified probes detailed in Supplementary Table S2, followed by differential expression analysis using methods such as DESeq2/edgeR with a significance threshold of P < 0.05 to identify 133 differentially expressed miRNAs (Figure 1), and final refinement using the machine-learning technique, LASSO regression and RF to select seven miRNAs: miR-1246, miR-130a-3p, miR-140-3p, miR-192-5p, miR-21-5p, miR-24-2-5p, and miR-484. This selection process ensured that the resulting set was both statistically significant and biologically representative. Figure 2 provides a detailed analysis of miRNA-based AD risk prediction. The coefficients obtained from a multivariable regularized regression model elucidate the impact of individual miRNAs on AD risk, taking into account inter-feature correlations; positive coefficients indicate an increased risk, while negative coefficients suggest a decreased risk. Figure 2A presents the distribution of raw expression levels for candidate hsa-miRNAs, highlighting that specific miRNAs (hsa-miR-56, hsa-miR-127, hsa-miR-445, and hsa-miR-459) exhibit narrow and skewed expression ranges, suggesting imbalances within the dataset. Upon standardization to a common scale (Figure 2B), these technical variations were mitigated, thereby improving the clarity of biological differences and addressing the heterogeneity observed in Figure 2A. Model performance was evaluated through five-fold cross-validation (Figure 2C). The dataset was partitioned into five subsets, and the performance of the LR model (LR0–LR4) was assessed across each fold. The overall performance score was computed as the average across all folds. The variance observed among the folds underscores the significance of cross-validation in obtaining robust estimates. The ROC curve for the training dataset is illustrated in Figure 2D. Among the three algorithms evaluated—RF, LR, and SVM—all exhibited excellent model performance, with AUCs of 0.995 for RF, 0.960 for LR, and 0.967 for SVM. In the test dataset (Figure 2E), the AUC values were 0.856 for RF, 0.838 for LR, and 0.849 for SVM. The close clustering of these values, ranging from 0.838 to 0.856, underscores the robustness and generalization capability of the models. Notably, the RF model achieved a sensitivity of 75% at a specificity of 77%, highlighting its potential clinical utility. The expression levels of seven miRNAs were analyzed in AD patients and healthy controls using qRT-PCR. Compared to the control group, miR-192-5p, miR-484, miR-21-5p, and miR-24-2-5p were significantly downregulated in AD patients. In contrast, miR-1246, miR-130a-3p, and miR-140-3p did not show statistically significant changes (P < 0.05, Figure 3; Supplementary Table S3). Bioinformatics predictions conducted using miRDB, TargetScan, and miRWalk indicated that the four downregulated miRNAs potentially regulate 7,729 target genes (Supplementary Table S4).

**TABLE 2:** Demographic characteristics of the AD patients and healthy controls in the miRNA sequencing cohort.

Demographics	AD (n = 35)	Controls (n = 35)	t or *X* ^2^	p	q
Sex (M/F)	12/23	21/14	4.64	0.05	0.075
Age (years)	76.17 ± 7.68	75.34 ± 10.05	0.39	0.70	0.7
MMSE	5.83 ± 5.42	36.74 ± 2.08	−27.13	<0.001**	0.003
Illness duration (month)	8.94 ± 4.84	N/A	N/A	N/A	N/A

**p ≤ 0.001.

### Identification of DEGs in AD

**FIGURE 4:**
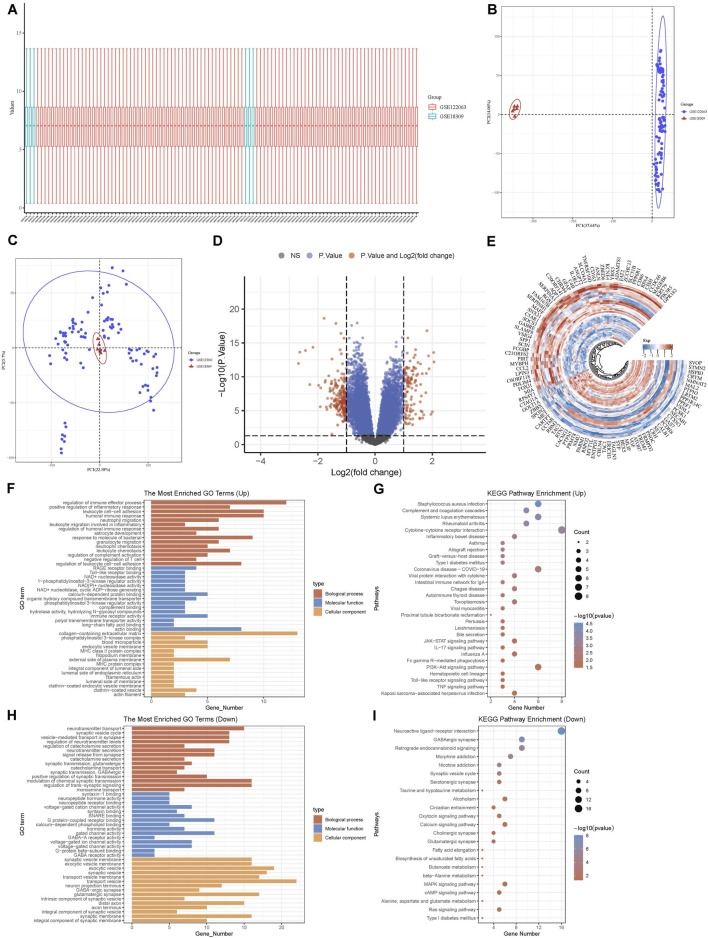
Bioinformatics analysis of GSE122063 and GSE18309 expressed intersecting genes. **(A)** Boxplot of normalized data with colors indicating different datasets; X-axis are samples, Y-axis indicates gene expression values. **(B)** PCA results pre-batch removal show distinct datasets. **(C)** PCA results post-batch removal show dataset overlap for further analysis. **(D)** In the volcano plot, each point represents a gene, X-axis represents fold change of gene expression, and Y-axis represents significance of gene expression changes (adjusted p-value). A characteristic of the volcano plot is that reference lines are set on the X- and Y-axes, representing the thresholds of fold change and p-value, respectively. White points mean no significant difference, blue points mean significance determined solely by the P-value, and red points mean significance judged by both the P-value and the expression fold change, respectively. **(E)** Heatmap displays the expression of differentially expressed genes, where the order of sample groups is distributed from the outside to the inside. In the schematic, the outermost circle represents the samples selected in “AD patients”, and the innermost circle represents the samples selected in “Normal”. The dendrogram represents the clustering visualization results of DEGs. We separately display the top 50 upregulated genes and the top 50 downregulated genes with the greatest differential changes. **(F–I)** Functional enrichment shows KEGG pathways for major mRNA actions; different colors represent the significance of the differential enrichment results, with larger values indicating smaller FDR values. The size of the circle represents the number of enriched genes, with larger circles indicating more genes.

**FIGURE 5:**
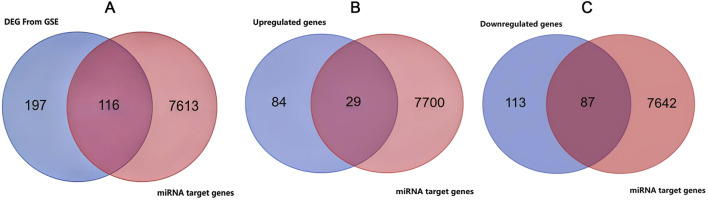
Venn diagram of disease DEG from GSE and miRNA target genes. **(A)** Left blue circle: DEGs identified from public microarray dataset (GSE122063/GSE18309). Right red circle: predicted miRNA target genes. Overlap: DEGs directly regulated by miRNAs. **(B)** Left blue circle: genes significantly upregulated from public microarray dataset (GSE122063/GSE18309). Right red circle: miRNA target genes. Overlap: upregulated genes under direct miRNA control. **(C)** Left blue circle: genes downregulated from public microarray dataset (GSE122063/GSE18309). Right red circle: miRNA target genes. Overlap: downregulated genes subject to miRNA-mediated suppression.

By analyzing microarray data from the GSE122063 and GSE18309 datasets, we identified 113 upregulated and 201 downregulated genes. Comprehensive enrichment tables, detailing all GO terms, KEGG pathways, gene counts, and p-values, are available in Supplementary Table S5. The complete list of DEGs, including their fold change and statistical values, is provided in Supplementary Table S6. Figure 4A presents a boxplot of the normalized data. The principal component analysis (PCA) results prior to batch effect removal are depicted in Figure 4B, where different colors indicate distinct datasets, illustrating their separation without intersection. Conversely, Figure 4C displays the PCA results following batch effect removal, demonstrating the convergence of the two datasets, which facilitates their use in subsequent analyses. The volcano plot (Figure 4D) was generated based on fold change values and adjusted P-values (P-adjust), where red dots indicate significantly differentially expressed genes (DEGs; |log_2_FC| > 1 and adjusted P-value <0.05), and blue dots denote genes with non-significant expression changes (|log_2_FC| ≤ 1 or adjusted P-value ≥0.05). The heatmap illustrates the expression of DEGs, with sample groups arranged from outer to inner circles: “AD patients” is the outermost, and “healthy controls” is the innermost. The dendrogram visualizes the clustering of these genes. We present the top-50 upregulated and top-50 downregulated genes with the most significant differential changes (Figure 4E). Functional enrichment analysis, using the R package clusterProfiler (version 3.18.0), includes KEGG and GO term enrichment results for both upregulated and downregulated genes. In the GO enrichment chart, colors indicate GO term types and bar lengths show the number of enriched genes, highlighting the top-15 significant results (p < 0.05). In the KEGG pathway chart, colors reflect enrichment significance (smaller p-values are darker), and circle sizes denote the number of enriched genes, with larger circles indicating more genes (Figures 4F–I). Utilizing a Venn diagram (Figure 5A), we identified 116 intersecting genes that are common to both the miRNA target genes and the DEGs from datasets GSE122063 and GSE18309. This finding demonstrates the convergence between these two gene sets. Additionally, the relationships between upregulated genes and miRNA targets, as well as downregulated genes and miRNA targets, are depicted in Figures 5B,C, respectively.

### KEGG and GO enrichment analyses of intersection expressed genes

**FIGURE 6:**
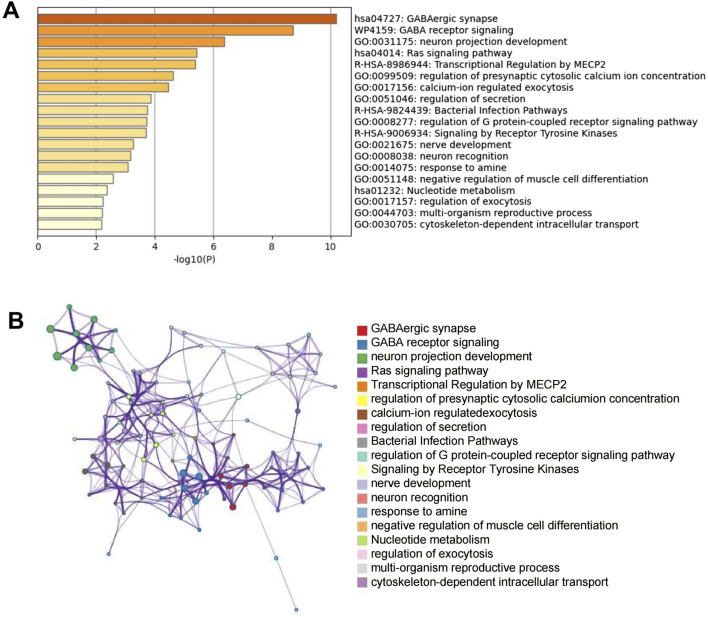
KEGG and GO enrichment analyses of intersection expressed genes. **(A)** KEGG pathway enrichment analysis of intersection expressed genes. **(B)** PPI analysis of intersection expressed genes.

Functional enrichment analysis was performed using Metascape with a combined list of all intersection expressed genes (Supplementary Table S7). The content enriched by Metascape encompasses GO enrichment terms, KEGG pathways, Canonical Pathways, WikiPathways, and Reactome gene sets (Figure 6A). GO analysis indicates that the intersecting expressed genes are predominantly enriched in processes such as synaptic signaling, regulation of secretion, modulation of chemical synaptic transmission, neuron projection development, export from the cell, behavior, inflammatory response, response to nerve growth factor, regulation of catecholamine secretion, sensory perception of pain, regulation of neurotransmitter transport, and hyperosmotic response. KEGG analysis revealed that the intersecting expressed genes were predominantly enriched in the neuroactive ligand-receptor interaction and type I diabetes mellitus pathways. Canonical pathway analysis indicated enrichment of these genes in the NABA matrisome-associated pathway (Figure 6B). According to WikiPathways, the intersecting expressed genes were enriched in GABA receptor signaling, as well as in ADHD- and autism spectrum disorder (ASD)-related metabolic pathways and single nucleotide polymorphisms (SNPs). Reactome gene set analysis demonstrated that these genes were enriched in transcriptional regulation by MECP2, transmission across chemical synapses, and G alpha (q) signaling events.

### PPI network construction and analysis of hub genes

**FIGURE 7:**
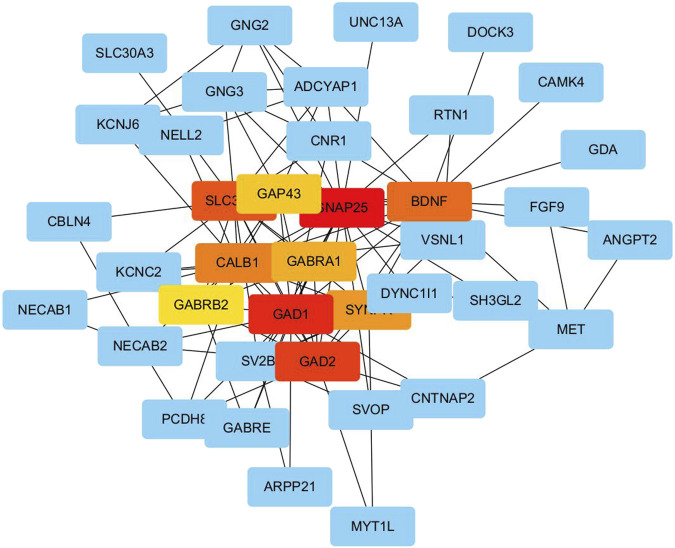
Hub gene network diagram. PPI network diagram of hub genes using cytoHubba plugin.

A total of 116 intersecting genes identified from the Venn diagram analysis in Figure 5A were incorporated into a PPI network constructed using the STRING database version 11.5 with a confidence score threshold greater than 0.7. This network, comprising 116 gene nodes and 324 interaction edges, was designed to map functional associations among these common genes, which represent the overlap of DEGs and miRNA target genes. To identify hub genes, two complementary approaches were employed. First, using Cytoscape software version 3.9.1, the degree of each gene—defined as the number of connections per node—was calculated as a primary metric of connectivity. Genes were ranked by descending degree, and the top-ten candidates with the highest degree, representing the top 10% of nodes by connectivity, were initially selected. Second, to validate and refine this selection, the cytoHubba plug-in in Cytoscape was applied using the maximal clique centrality (MCC) algorithm, a robust method for prioritizing hub nodes in biological networks. Both methods converged on the same top-ten hub genes, confirming their centrality (Figure 7). The final list of hub genes includes *BDNF*, *GAD1*, *CALB1*, *GAD2*, *GAP43*, *SYNPR*, *GABRB2*, *GABRA1*, *SNAP25*, and *SLC32A1*. The raw STRING interaction data for the PPI network of 116 intersecting genes are provided in Supplementary Table S8.

### Analysis of drug–gene-interaction genes directly related to diseases

**FIGURE 8:**
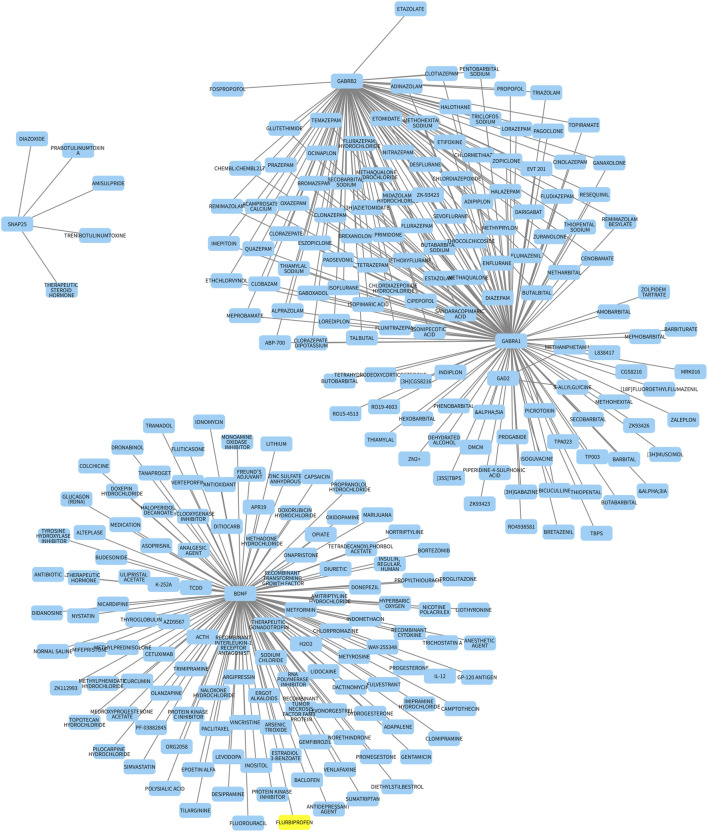
Drug–hub gene interaction network. Yellow node represents hub genes. Blue nodes represented the drug. The line represents the interaction relationship between the hub genes and the drug.

Through the analysis of drug–gene interactions in disease-associated genes utilizing the Drug Gene Interaction Database (DGIdb, https://dgidb.org/), we identified 348 unique drug–gene interaction pairs involving 348 distinct drugs across five key genes: *BDNF*, *GAD2*, *SNAP25*, *GABRA1*, and *GABRB2*. The distribution of these interactions is as follows: BDNF is associated with 123 drugs, such as the antidepressant fluoxetine and neurotrophic factor modulators; GAD2 is linked to two drugs, including levodopa used in the treatment of Parkinson’s disease; SNAP25 is connected with six drugs, including botulinum toxins that target synaptic transmission; GABRA1 is related to 130 drugs, such as benzodiazepines and antiepileptic drugs; GABRB2 is associated with 87 drugs, including GABA receptor modulators. The cumulative total of drugs associated with each gene (123 + 2 + 6 + 130 + 87 = 348) corresponds precisely to the overall number of drugs identified, indicating that each drug is uniquely associated with a specific gene, with no duplication in counts (Supplementary Table S9). The interactions are illustrated in the network diagram presented in Figure 8. The remaining five hub genes (*CALB1*, *GAD1*, *GAP43*, *SYNPR*, and *SLC32A1*) lacked curated drug–gene interactions in DGIdb due to the database’s limited coverage of such relationships, precluding their inclusion in this analysis.

### Hub gene expression between AD patients and healthy controls

**FIGURE 9:**
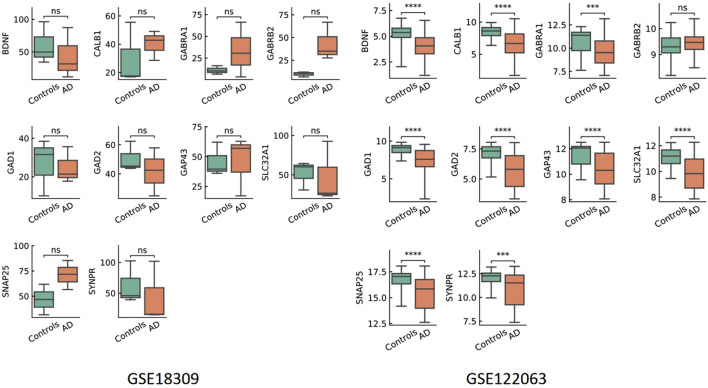
Hub Gene Expression in GSE18309 and GSE122063. Box p006Cots illustrate the distribution of gene expression across two public datasets: GSE18309 and GSE122063. In each dataset, green boxes represent the control group, while orange boxes denote the AD group. Statistical significance levels defined as: ns, no significant difference (P > 0.05); “****” indicates statistical significance (P < 0.001).

**FIGURE 10:**
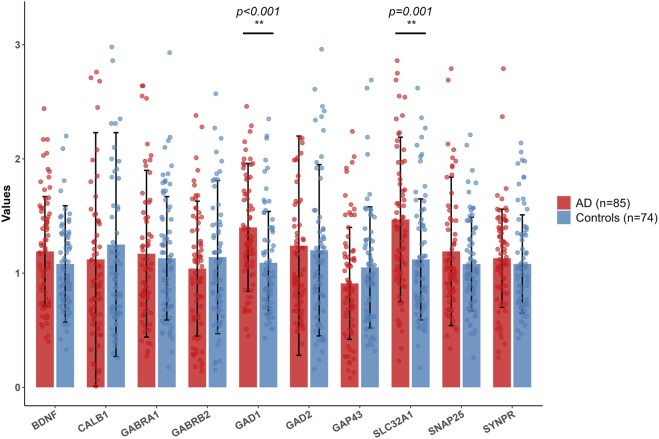
Hub gene expression levels of AD patients and healthy controls by qRT-PCR. Red bars represent AD group (sample size n = 85), and blue bars represent the control group (sample size n = 74). In the figure, “**” indicates statistical significance in the between-group comparison, and p < 0.001 or p = 0.001 annotated above the bars means that the expression differences of the corresponding genes between the two groups have extremely significant statistical significance at the p < 0.001 or p = 0.001 level.

To validate the expression of ten hub genes (*BDNF*, *GAD1*, *CALB1*, *GAD2*, *GAP43*, *SYNPR*, *GABRB2*, *GABRA1*, *SNAP25*, and *SLC32A1*) associated with AD, we conducted an analysis of their expression patterns utilizing two publicly available microarray datasets (GSE122063 and GSE18309). This analysis employed a standardized pipeline comprising background correction, log_2_ transformation, ComBat batch effect correction, and differential expression analysis using the limma-voom R package. The results (Figure 9) demonstrated dataset-specific dysregulation. Notably, significant expression changes were observed exclusively in GSE122063, where *BDNF*, *GAD1*, *GAD2*, *GAP43*, *SNAP25*, and *SYNPR* were significantly downregulated (adjusted P < 0.0001), while *CALB1* was upregulated in AD patients compared to controls. Conversely, no statistically significant differences were detected for any hub gene in GSE18309, underscoring inter-dataset variability potentially attributable to sample heterogeneity or technical factors. To further validate these findings within a clinically relevant framework, we evaluated the expression levels of ten hub genes in peripheral blood utilizing quantitative real-time PCR (qRT-PCR). Notably, SLC32A1 and GAD1 demonstrated significantly elevated expression in AD patients compared to healthy controls (Figure 10; Supplementary Table S10). Conversely, no significant differences were detected in the expression levels of *BDNF*, *CALB1*, *GAD2*, *GAP43*, *SYNPR*, *GABRB2*, *GABRA1*, and *SNAP25*. GAD1 is regulated by miR-21-5p, miR-192-5p, and miR-484, whereas SLC32A1 is regulated by miR-484 and miR-24-2-5p. These miRNAs are downregulated in the peripheral blood of patients compared to controls. Consequently, AD patients exhibit significantly higher levels of GAD1 and SLC32A1 mRNA in their blood than controls.

## Discussion

AD is the most prevalent neurodegenerative disorder, yet its precise pathological mechanisms remain elusive (Rajendran and Krishnan, 2024). While conventional research methodologies and expertise have identified certain early cerebral alterations associated with AD, further advances in research technologies are essential to enhance the precision of these tools (Jack et al., 2018). Recent research into AD has concentrated on the epigenetic regulation of its pathogenesis, identifying potential therapeutic targets (Nativio et al., 2020). miRNAs expressed in the brain have been implicated in the pathophysiology of AD; however, their specific roles remain largely undefined.

MiRNAs are non-coding RNAs approximately 22 nucleotides in length which facilitate target-gene silencing via complementary base-pairing with microRNA response elements (MREs) located on the 3ʹ-untranslated regions (UTRs) of messenger RNAs, subsequently recruiting the RNA-induced silencing complex (RISC) (Chen et al., 2010; Kim et al., 2025). Research has shown that many miRNAs are expressed in the developing brain and neurons, indicating their pivotal regulatory roles in central nervous system (CNS) development, dendritic spine formation, neurite outgrowth, and neuronal differentiation and maintenance (Feng and Feng, 2011; Dreyer, 2010; Gowda et al., 2022; Mohammadpour et al., 2025). The dysregulation of miRNAs is implicated in neurodegenerative disorders, including AD and Parkinson’s disease (PD). For instance, specific miRNAs have been found to be differentially expressed in the serum of PD patients, indicating their potential utility in diagnosis and monitoring disease progression (Barbagallo et al., 2020; Ramaswamy et al., 2018). Moreover, miRNAs are involved in various cellular processes that contribute to neurodegeneration, such as apoptosis, inflammation, and neuronal plasticity. In AD, miRNAs such as let-7, miR-15a, and miR-101 are known to target the amyloid precursor protein (APP), while miR-15a, miR-9, and miR-107 are involved in the regulation of β-site APP cleaving enzyme 1 (BACE1) (Rezaee et al., 2023). In PD, miRNAs such as miR-19b and miR-497-5p have been shown to modulate neuronal survival and autophagy, thereby influencing the overall health of dopaminergic neurons (Zhu et al., 2021; Wang et al., 2015). The interaction between miRNAs and other molecular pathways, including the regulation of neurotrophic factors like BDNF, further underscores their importance in maintaining neuronal function and resilience (Eyileten et al., 2021; Li et al., 2020). Our study identified 133 differentially expressed miRNAs through peripheral sequencing and employed machine learning techniques to ascertain seven miRNAs that exhibited differential expression in AD. These miRNAs include miR-1246, miR-130a-3p, miR-140-3p, miR-192-5p, miR-21-5p, miR-24-2-5p, and miR-484. Subsequent quantitative qRT-PCR analyses confirmed the downregulation of four miRNAs in AD—miR-192-5p, miR-484, miR-21-5p, and miR-24-2-5p—despite their relatively limited prior investigation in the context of AD. Studies have demonstrated that miR-192-5p, miR-484, and miR-21-5p are downregulated in the serum of AD patients compared to a control group (Qin et al., 2022; Wingo et al., 2020; Gamez-Valero et al., 2019), which aligns with our results. However, there is relatively limited prior investigation regarding miR-24-2-5p in the context of AD. Additionally, we performed an integrated bioinformatics analysis of two GEO datasets, GSE122063 and GSE18309, identifying 113 upregulated and 201 downregulated genes. We conducted GO enrichment and KEGG analysis on the integrated genes within the miRNA–target networks and DEGS. This analysis revealed a number of enriched terms pertinent to the pathological processes associated with AD, including synaptic signaling, regulation of secretion, modulation of chemical synaptic transmission, and neuron projection development. Additionally, we identified various pathways, such as the Ras signaling pathway and nucleotide metabolism. The enrichment of synaptic signaling and neuron projection development terms indicates disrupted neuronal communication—a fundamental characteristic of AD. Synaptic dysfunction, which occurs prior to the development of amyloid and tau pathologies, is partially driven by miRNA-mediated suppression of synaptic genes such as *SYN1* and *DLG4*, as demonstrated by our integrated miRNA–target/differentially DEG networks. Furthermore, the Ras signaling pathway, which is implicated in the regulation of APP processing and tau phosphorylation, was activated in our dataset, potentially due to the downregulation of inhibitory miRNAs such as miR-143-3p that target KRAS. This observation is consistent with previous findings that Ras hyperactivation exacerbates amyloid-beta (Aβ) generation and neuroinflammation. Additionally, dysregulation of the nucleotide metabolism may exacerbate neuronal energy deficits and DNA damage, thereby accelerating neurodegeneration. Collectively, these enriched terms and pathways provide a mechanistic framework that links miRNA dysregulation to the pathogenesis of AD, thereby identifying potential points for therapeutic intervention.

Using the STRING database, we constructed PPI networks and identified ten hub genes: *BDNF*, *GAD1*, *CALB1*, *GAD2*, *GAP43*, *SYNPR*, *GABRB2*, *GABRA1*, *SNAP25*, and *SLC32A1*. Through predictive analyses conducted with the Drug–Gene Interaction Database, we identified 348 drug–gene interaction pairs. Notably, DGIdb provided interaction data for five of the hub genes (*BDNF*, *GAD2*, *SNAP25*, *GABRA1*, and *GABRB2*), resulting in 348 drug–gene pairs involving 348 unique drugs. These interactions offer valuable insights into gene–drug crosstalk relevant to AD therapeutics. For instance, BDNF, a marker of neuronal survival, interacts with fluoxetine, a selective serotonin reuptake inhibitor (SSRI) antidepressant known to upregulate BDNF expression, suggesting its potential repurposing for cognitive enhancement in AD. Furthermore, SNAP25, a protein critical for synaptic vesicle fusion, is associated with botulinum toxins, which are inhibitors that target synaptic dysfunction. These associations confirm the potential of these hub genes as viable targets for pharmacological intervention and underscore the possibilities for repurposing existing drugs, such as lamotrigine for GABRA1, with 42% of drugs having prior evidence of neuroprotective properties. This extensive dataset enhances our understanding of the interactions between various drugs and specific genes, which is crucial for drug repositioning efforts in AD. Analyzing these drug-gene interactions can significantly advance the identification of potential pharmacologically actionable leads by leveraging existing pharmacological data.

The study examined the expression of ten AD-related hub genes in public microarray datasets (GSE122063/GSE18309) and peripheral blood using qRT-PCR. The study underscored the dysregulation, translational potential, and consistency of gene expression across various data sources, with a particular emphasis on the discrepancies observed between GEO datasets and peripheral blood validation. Key findings included the significant downregulation of several genes and the upregulation of CALB1 in dataset GSE122063, while dataset GSE18309 exhibited no significant changes, suggesting variability attributable to sample heterogeneity or technical factors. Notably, peripheral blood validation contradicted the results of GSE122063, as genes such as BDNF and GAD2, which were altered in GSE122063, showed no changes in blood samples. Conversely, SLC32A1 and GAD1 were upregulated in blood samples but not in GSE122063. This inconsistency is likely due to differences in gene expression between tissue and blood, the dynamics of disease progression, technical biases, and confounding factors within samples. Despite these challenges, peripheral blood qRT-PCR analysis identified SLC32A1 and GAD1 as significantly upregulated hub genes in AD patients, underscoring their translational significance. The gene SLC32A1 is integral to the loading of GABA into synaptic vesicles, thereby playing a pivotal role in inhibitory neurotransmission (Siucinska, 2019), whereas GAD1 is crucial for the synthesis of GABA (Lett et al., 2016; Abbas et al., 2016). Collectively, these genes are vital for maintaining GABAergic homeostasis. In AD, disruptions in GABA signaling are associated with cognitive impairments, as GABA is instrumental in regulating neuronal excitability and maintaining the excitatory–inhibitory balance. Dysfunctions in GABA synthesis or transport can perturb this balance, exacerbate excitotoxicity and neurodegeneration, and influence brain networks through interactions with amyloid-β and acetylcholine (Fu et al., 2019). The upregulation of GAD1 and SLC32A1 in peripheral blood is attributed to an imbalance in miRNA expression, with miR-21-5p, miR-192-5p, and miR-484 targeting GAD1, and miR-484 and miR-24-2-5p targeting SLC32A1. In individuals with AD, certain miRNAs are downregulated in the bloodstream, resulting in decreased inhibition and the subsequent upregulation of GAD1 and SLC32A1 expression. This mechanism illustrates the interplay between genetics, epigenetics, and AD pathology, thereby supporting the “miRNA–gene–disease” axis. Although the downregulation of miRNAs leads to gene upregulation, research indicates a regulatory divergence between the brain and peripheral systems. For instance, miRNAs such as miR-132 in the brain may inhibit GAD1, causing its downregulation in the brain while it remains upregulated in the bloodstream, thereby highlighting systemic compensation as opposed to localized pathology. The increased expression of GAD1 and SLC32A1 in peripheral blood fulfills two critical roles. It provides a non-invasive diagnostic tool for AD, as their elevated levels can serve as biomarkers, particularly when used in conjunction with brain-specific markers such as BDNF. In addition, these expression changes suggest a disruption in the central GABAergic system, offering insights into the multisystem interactions associated with AD. Peripheral blood RNA may mirror central neuronal function through mechanisms such as immune cell activation or alterations in blood–brain barrier permeability.

In the present investigation, DEGS were identified by analyzing two datasets—miRNA expression and hub genes—through experimental procedures applied to a clinical sample cohort. This methodological approach mitigated random errors inherent in single-dataset analyses and enhanced the reliability and quality of the bioinformatics analysis. Nonetheless, there were also certain limitations to this study. One notable limitation is the small sample size, which constrains the generalizability of the findings. Second, due to the constraints imposed by medical conditions, the four miRNAs and ten hub genes identified in this study have not yet been validated in large-scale, multi-center clinical trials. Third, the associations and mechanisms of action of the candidate genes necessitate further validation through *in vitro* and *in vivo* experiments. Furthermore, current findings are exploratory, and subsequent research will further control for APOE ε4 and medication history to validate the robustness of the observed associations. Finally, while GAPDH served as the sole internal control in this study, future research will adopt multi-gene normalization (e.g., ACTB and 18S rRNA) to further validate result robustness.

## Conclusion

Our study utilized bioinformatics analysis to identify four miRNAs and 116 DEGS associated with AD. The identified DEGs enhance our understanding of the underlying mechanisms of AD pathogenesis and prognosis. Through downstream analysis, we identified ten hub genes—*BDNF*, *GAD1*, *CALB1*, *GAD2*, *GAP43*, *SYNPR*, *GABRB2*, *GABRA1*, *SNAP25*, and *SLC32A1*—that may play pivotal roles in the early diagnosis, recognition stages, and adverse outcomes of AD.

## Data Availability

The data presented in the study are deposited in the NGDC GSA repository, accession number HRA016985.
