# Supplementary material for: miRNA-associated gene networks reveal potential candidate markers for Alzheimer’s disease
Source: Front Mol Biosci. 2026 Mar 6;12:1699404. doi: 10.3389/fmolb.2025.1699404 (PMC13002409; doi:10.3389/fmolb.2025.1699404)
Supplement: Supplementary file 1 [file Supplementaryfile1.zip › Supplementary Tables/Supplementary Table 3.docx]

**Supplementary Table 3. miRNA expression levels of AD patients and healthy controls by qRT-PCR.**

|  | AD (n=85) | Controls(n=74) | z | *p* | *q* |
| --- | --- | --- | --- | --- | --- |
| miR-140-3p | 0.94±0.78 | 1.17±0.75 | 0.28 | 0.78 | 0.800 |
| miR-192-5p | 0.65±0.41 | 1.21±1.06 | 3.32 | 0.001** | 0.005 |
| miR-484 | 0.74±0.67 | 1.03±0.77 | 2.67 | 0.008* | 0.032 |
| miR-21-5p | 0.96±0.85 | 1.17±1.04 | 2.05 | 0.04* | 0.114 |
| miR-24-2-5p | 1.20±1.09 | 1.57±0.85 | 2.13 | 0.033* | 0.110 |
| miR-1246 | 1.35±1.12 | 1.28±1.09 | 0.5 | 0.615 | 0.724 |
| miR-130a-3p | 1.07±0.65 | 1.11±1.01 | 1.55 | 0.122 | 0.244 |
