# Supplementary material for: miRNA-associated gene networks reveal potential candidate markers for Alzheimer’s disease
Source: Front Mol Biosci. 2026 Mar 6;12:1699404. doi: 10.3389/fmolb.2025.1699404 (PMC13002409; doi:10.3389/fmolb.2025.1699404)
Supplement: Supplementary file 1 [file Supplementaryfile1.zip › Supplementary Tables/Supplementary Table 4.docx]

**Supplementary Table 2**

**miRNA target gene**

| miR-24, miR-192-5p, miR-484, miR-21-5p target genes |
| --- |
| DPM2 FSTL1 CLMP AACS ELMO2 SLC18A1 SLMAP SAMD4A HAUS2 FARP1 PAX7 KCNMA1 C10orf90 ZHX3 FAM153A ERCC5 FAM187A RXFP3 APBB2 MEFV XDH ABCB7 ABCC6 GFOD2 AEN SLC25A3 SLC10A7 BRIX1 PXYLP1 ESYT1 LCORL LDB3 IFT122 CHD8 GP1BA ANGPTL5 OR7A10 SLC6A11 CHRNA10 TOP1MT MYO9B CRNKL1 NTSR1 GPSM2 CPXM2 KLHL14 HIC2 NOS2 MON1B TRIB3 SLC2A5 DDX5 C2orf49 ZEB1 ZG16 CDK14 ERG PARN SOD2 HROB DIRAS1 PNPT1 LRMDA MIER1 CCDC28B KCNK3 MSRB3 PDE1C SEMA4D HEPH TOB2 NOSTRIN ING3 TBC1D3H OR2V2 MAP3K3 COPS7A MAX GRM2 RNF185 OSBPL8 TNS3 LRRN1 IPO9 C4orf3 PCARE RRP7A ZFYVE1 CXCL13 SCYL3 TEAD1 SNX33 NFIA ARL6IP1 CHD1 TMEM184A TCEANC2 RPS6KA5 MAPKAPK2 ATXN10 NMNAT1 LHFPL2 DIP2A CCL22 CCDC34 CMPK2 SLC12A4 BRCA1 CDC25B BMP3 LPAR4 TMEM19 MAP1LC3C RUNX3 SLK LUZP1 EFNB1 PRKCB ACSM3 CNBD2 ATP5MG NOL8 USP6NL ABCD4 VKORC1 TEK LARP7 CD160 FOXK2 RNF32 SLC8A1 GTPBP1 ATL2 AP1S2 DNAAF10 RAB33B TMEM266 RBM17 ZNF212 PRDM2 SMIM35 SUPT4H1 PDCD4 SRRM4 EOLA2 FOXP3 OR2J1 NUP42 RAPGEF6 RNF223 NEIL1 ANO5 ANKRD6 DCAF4L1 KCNC1 SIRT6 OR8J1 ARHGEF9 STARD5 FAM98A PGAP4 ABR C2CD3 CD38 CHRAC1 PGBD5 CTDSP2 RMND5B DUSP4 CCNY SSU72 SMUG1 AP3M2 USP14 MAOA CORO2A ETNK2 DSTN FBXO31 FRS2 ST8SIA4 GASK1B RNF4 EMC1 ING5 ALG2 PPP1R3D PLEKHG4 GRB2 TPM3 ATXN7L3B DUS3L LCOR CHST15 CETN3 PRY N4BP2L2 KLHL36 OPHN1 ADH5 P2RX3 MARF1 CDIPT TOR3A PMF1 C8orf44-SGK3 HS2ST1 CD82 MAP3K13 SETDB1 GCLM EIF1AX PPM1A CPEB3 BCL2 MMP16 PGGHG MYO15B PTP4A2 AIPL1 IL24 SNX19 WDFY2 CHFR THADA TAF12 ID1 PIK3CB TNXB ELAPOR2 FAXC SMIM27 FAM110C ZNF35 LIMD1 CARD8 TF RSRC1 CLCN4 CHST11 THRB ELAC2 AQP2 SLC9A9 CFAP221 GSTM4 EMC8 SEC23IP RTL3 RCSD1 SNX6 ZBTB11 POLG TFR2 B3GALT5 SPTBN1 RICTOR RAG1 PRR22 CDC42EP3 SRP72 TSPAN7 OR4D10 MFN2 IDO2 RHOH NUCB1 ATR FSIP1 CAPN1 DDHD2 FAM204A UPF3A SNX25 GNRHR CHST6 UNC13B ZNF687 PPBP SDC2 SLITRK4 MAPK10 KDM2A USP32 NCAM1 PDP1 ABHD12 AHCYL2 GLIS2 DAPP1 ATF7 PEX16 LRRC10 TARS1 CNTN2 TKTL1 FGF1 IL6R UBN2 PRR27 CAPN12 DNAJA3 GLG1 CIITA EPHA4 MUTYH ZNF70 L2HGDH HGH1 GPATCH2 TMEM11 FAM234A MRPL15 KCNQ5 PLCXD2 AGPS ZBTB20 RPUSD3 NEU1 MED14 HP1BP3 PCCB ENHO SPRY2 MRM3 LZTFL1 DCUN1D5 HDHD2 GMFB TDRKH IGDCC3 GNB4 ANKLE2 NBPF3 CEP85 PCSK6 LILRA6 RPRD1B PRR23D2 CCDC172 WASHC3 DNHD1 ASAH1 LONRF2 CIP2A NFYA NRG4 UBE2W CFAP92 ACTR5 IL18BP SCN11A FHL1 TRMT6 PSMD5 HK3 MYLK2 E4F1 IRAG2 ACO2 KCNA4 PALM2AKAP2 SORBS2 PSMF1 NFE2L2 SH3PXD2A SH2D2A KLF4 IFITM10 LMO4 ENTR1 SCHIP1 JADE1 HP TRAF7 TAGAP SYTL5 MRPL57 IDS PARG DNAJC30 C1orf112 DYRK4 HAPLN4 MICALL1 UIMC1 ZNF667 PTGES3L-AARSD1 TFEC ATP7B POLD2 PPM1H DCX KIN PPARGC1A DUS4L RTEL1 ZNF709 KCNK10 CASP5 RNF182 OTOA OR6A2 CHMP6 AHRR PDAP1 BRD4 ZNF146 MIA2 DOK4 ARFRP1 EIF4E2 HRH2 HOXB5 MTMR6 TTC13 ENTPD7 CYP3A5 CUL4B CENPJ TNRC18 FAU PRR36 FHDC1 LDLRAD2 RDX OMD SLC4A5 DNAJC9 ITSN1 OAS1 WARS1 ADCY3 CAMK2B LIN28B TMEM241 TTC3 GRHL1 SRMS SLA DLST MYCBPAP ANKRD26 ATP2B2 VWCE CFC1B LXN TLK1 TBC1D3B MTMR12 PREX2 ESPNL LDLR KLHL21 ASTN2 PI15 RER1 PROSER2 TAB2 GPATCH8 KIAA0930 HPS1 RNF7 KIF4A FAM161A TFE3 PGM1 ZNF586 SNCAIP KCTD13 ANXA8 CSMD3 DLGAP1 FBF1 XKR9 IRF7 SET FBXL20 PECR HSPA2 FAM120A SLC38A9 PCDHGA10 SERP1 SHH THPO EIF2S1 CD177 PIGG JAK2 TMPRSS4 C1orf115 BICD1 TNFRSF1A SHISAL1 DCAF11 ACADVL ISCU GTF2H5 XAF1 MYCL TCP11X2 HBP1 TNNT2 ABCG8 PHGDH XPO4 XIAP OTUD7A TLX1 APOA2 KALRN VAX1 ZDHHC22 GPR174 THOC7 OR8K3 IFNAR1 ATP6V0A2 KIAA0319L SLC29A3 RAB11FIP4 NF1 C6orf141 OLR1 TAOK2 DAGLB SEPHS1 PPFIBP1 NEGR1 NCF1 SPG7 MCM10 MAP4K3 RMDN3 IQANK1 HOXA5 CLK3 NPIPB5 MICA INPP5B TRIM56 HNRNPK TCFL5 IGLL5 PPCDC BTBD9 NT5E FOXN2 NCSTN FPGT-TNNI3K SAFB2 GK5 SC5D SLC9A2 SULF2 CD93 NEURL3 ADD1 ZNF136 DNAJC1 CCDC144A ACLY HPCAL4 CYP4F12 TRAIP MARCHF4 LCN9 KIAA0232 FKBP5 ERCC8 RRAGC NFAT5 FBXO3 CMKLR1 TMOD2 OR52A1 FAM199X CTAGE4 DGKQ NOL4L ALG9 CPA4 HERC3 ZBTB47 PTP4A3 CUL3 PODXL ANKRD40 SNCA GPX2 ITK CLIC5 KMT2B ZNF551 ESRRB NR1D2 ARHGAP26 PRCD JAGN1 BSDC1 RBP7 RABL6 METTL16 DSC1 ZNF536 PTPN4 STX5 CTCFL EPB41L3 TEKT2 WSB1 PARVB CSRNP1 APOL1 SETBP1 RTP1 CDH11 RNF175 GIMAP6 DMBX1 ZBTB41 NRSN1 ITGA2 ACTRT3 VAT1 SENP3 PCNX2 GRIN2A MAST4 CCDC14 CAVIN2 TUFM SGO1 TRMT2B NRXN1 KRT34 NR4A3 SPDYA YJEFN3 DTWD2 UNKL C22orf23 IL10RB DNASE1L1 MACIR C19orf44 CHPF2 ALPK1 ARID1B ZFP1 E2F7 WDPCP VPS50 VCP MAGI1 MFSD14B SLC14A2 APOBEC3F SLC30A2 LZTS3 SRSF3 SP4 ICAM2 RALB KIF1B GPD1L VPS37B AMOTL2 MOSMO C6orf120 PSRC1 PIGT FOXO1 CBLN2 ADAMTS17 MATN4 PPP1R17 CRLF3 CLCNKB AMPD3 PIAS1 TSEN54 ZNF611 ZNF3 GRAMD2A TBC1D3 LRRFIP1 HLA-DQA1 GCLC ZC3H14 HMGXB4 MAN1A2 ALDH5A1 SEC61A1 MROH5 FKTN TWSG1 CTDP1 CRTAP ADAMTS6 TK2 IL18RAP SETD3 GALNT10 PRRX2 PRELID2 ELK4 CFAP74 MMP24 RHO GOLGA8J GRID1 SMURF2 SRL CR1 RPS6KA2 SLC22A3 HMBS FAM13A EFR3B ACER3 ADAMTS12 KLHDC1 ZNF287 AVL9 ARHGAP8 FOXG1 TCTN3 WFS1 TBC1D16 RAD21L1 OR2AG1 DNAJB11 CC2D1B SLCO1C1 TBC1D3I ATL1 PTCD2 ADGRG1 ASPN GRAMD1C ADGRF5 USP38 SERPINB11 ZDHHC17 NDE1 CDK18 PPARD ACSM6 KDM4A PHAX ACSS3 CLNK TBC1D24 ABCD3 CFAP70 DCDC1 VPS26B MTF2 DNAJB13 DGCR8 PARVG MUC22 GSR CACNA1C FIG4 PDCD6 FAM3C KCNH3 CCSER2 PLBD2 EIF2AK2 CCL11 ASB4 ALDH1L1 CFAP47 SLC12A7 RBM4B FCGR2A BMF RBMY1A1 PRKN STARD8 TGIF2LY NKAIN1 SIAH2 ATP11B FBXO34 CCDC103 RB1CC1 POLR3A ABHD5 EXOC4 TMBIM1 RMND5A TCHP HEATR5A TRMT1 EXOC3L4 KMT2D IMPG2 HTR4 SLC35F5 TRAF6 CCL18 PNPLA3 SPAG5 KIAA0408 CD276 F13A1 CSNK1E ACADM KRT72 XPO6 PIK3C2B OGN FMR1 KDM7A BBS9 USP15 C9orf72 ZPR1 RNF31 FNDC9 APELA SLC44A3 PTGER2 ERCC4 SLC31A1 RAD23A ACY1 IFT172 GFOD1 CTNNA1 CNTD1 C9orf153 CBX4 MYO9A BLMH TXLNG NTRK2 PFKFB2 TAS2R4 KIAA0513 SH3BP2 SH3TC2 WDR33 PRMT5 IRX4 GOLGB1 DCAF4 LCLAT1 SPANXN1 GOLGA8Q KCNS2 CHD7 WDFY3 SLC30A8 CEP63 GPSM1 HIC1 TLE3 LRP1 SHQ1 C19orf18 RFT1 TACC1 SLC6A1 SAMHD1 PTBP3 OCLN ARL17B SERTAD4 NELFA DDI1 MYSM1 CFAP91 CDON MMP14 TMEM38A SNX18 NLK MDM2 KCNK9 TDRD3 NETO1 KLHDC3 THSD7A NDST3 KLHL15 EFHD2 NBEA ABCA6 CXCL2 GNAI1 AKT2 EGFR VPS4B GOLGA6B ZDHHC3 SLC25A4 MAP3K15 CYB5B TESPA1 SESN1 PTPRQ TNPO1 CARD10 ZNF285 FARP2 SNX5 RASGEF1B PLGLB2 MAPKAPK3 NCAM2 PAPOLG CDK13 CHML ITPRID2 SIRT5 MSRB2 BCR DZIP1 SEC22C GPR161 FXYD5 RNF146 NKX3-1 CHD2 ZNF10 PURA DNPEP TBC1D3G ERBB2 CREB1 ADAMTS8 LNPK NEO1 MXRA7 CFL2 LRRTM2 STAG1 OSBPL7 LAT NECTIN4 KCNA3 IQCJ-SCHIP1 PIF1 TARS2 ABCF1 APOOL SMARCA4 ARMC9 TGFBRAP1 MASP2 FAHD2A CNTN1 ZBTB21 AGL NFE2L3 BBS4 TMEM181 PSD4 RGS11 CCL3L1 VSIG10L2 TNP2 AKR1D1 DNAI7 TTC39B STAT5B SKI PLEKHB2 ADGRG2 RTRAF OAS2 TRIM11 GLIS3 CAPN11 BIN2 ATXN7L3 TBL1XR1 FAIM SZT2 ANKRD36 MUC17 KLF5 DDIAS TNFSF8 DNAJC5B PPP2R2A EIF3J TOM1L1 ROBO4 TMEM167A GOLGA8K NRG3 F10 SNX11 LPCAT4 THBS1 ACSL1 ARMCX1 HCFC2 ABL1 PTPRG PLEKHG5 SNAPC5 NCOR1 MOCOS PRDM10 HEATR9 CMTR1 SRGAP1 SLC16A4 FAM156A CALCR CDC45 MBTD1 DCAF4L2 C16orf72 ELMO1 PPP1CB MYDGF ERBIN MAP3K12 SCIMP ARF5 SLC24A4 CCT3 SLC5A10 TARBP2 IER5 MBTPS2 CMTM4 MVK RBM5 KHDC4 RBMY1E TSPYL1 NCL UVSSA MOBP APLN IRAG1 WDR44 RASGRP3 PRKRIP1 GDF11 ADH4 WDR27 MCM2 TMEM200C MED21 CDAN1 MCM4 BTBD18 SPTBN2 PDZD2 MCC IFT88 CEP192 SLC26A2 ATP2B4 KIAA0895 KANSL3 CHST12 RAB22A SLC24A1 EOLA1 ATXN1L CHMP1A LRSAM1 B3GALT6 ZNF688 HDAC11 VMO1 PEDS1 MTO1 DHX29 ADAR MPIG6B BAHD1 PLXNB2 P2RX1 DNAH10 PDE10A METAP2 EN2 STXBP4 CCDC85C TOX2 MED15 HEY1 TBC1D32 KNG1 CASR GPAM UNC50 ESYT2 IRF2 VSTM2A RUNX2 PGR FCRL5 ZNF99 GRK4 PPP2CA FGF10 ODC1 MBNL3 SDC3 ZBTB8B SEPTIN3 PHEX SOX6 POLE THRA POLH MAPK11 TBC1D22A HIP1R PLEKHA2 PDE4DIP EMC6 RASGRP4 PEG3 NADK TRMT10A SGSH TRPM3 ZNF263 ZMYM4 STK32B RAP2A ATP6AP2 SLC7A7 ABHD10 CA8 SLC35C1 NPIPB4 LAMTOR4 RSPRY1 VCAN POLR2J3 CYP27C1 ZDHHC21 MED12L ERO1B PANK2 ASH2L CNTNAP5 TM9SF3 TCP11X1 TAF8 SLC1A3 DDHD1 ZNF594 BCL7B MAN2B2 SHPK RNH1 PPP6R3 MAFG BICD2 CDH7 TOMM5 SHANK2 RAB40C C2orf68 PRRG3 ACOXL LRCH1 FAM124A CLIP3 MAP4K2 ACAP2 FGF13 PRDM5 PPP4R2 ACP1 CDC23 PBXIP1 CASKIN2 ZMYM2 TAB3 GUCD1 ILDR2 C13orf42 DAGLA SOHLH1 ZNF550 SLC2A4RG LPL FTCDNL1 MIS12 UNC5D TRIML2 CUL7 DIO1 EIF2B1 HS3ST2 TGM4 SLC6A12 CD84 TENT4A RNF10 ZNF830 EVA1A NONO CLEC5A ITCH MGAT3 NCBP3 ARPC5L KLHL42 CENPB MBP KLHL26 GTF2H2C GJC1 TANGO6 SBSPON TBRG1 PIK3R1 LRRC74A SV2A PDGFRA GSG1 DOK5 HELZ SLC46A3 TET1 MPEG1 DMD STAMBP ADAT1 ARHGAP36 ADCY2 CENPI NEXMIF FOXH1 ANKRD33B NOVA1 OTULIN NMT1 SOCS5 TMIE CDH19 SSH3 LDLRAD1 ZNF135 CCBE1 CHURC1 TAAR1 PLEKHF1 DLG5 CCDC42 PGM5 PER1 TFEB TMEM272 ZFYVE27 SHTN1 ELMOD3 C10orf105 SMARCAD1 CNIH1 ATG12 SH3RF1 FNDC3B RNMT MTCP1 PROX2 MASP1 LMCD1 JAKMIP3 GPR63 BLZF1 SARAF SWI5 RPAP2 ATRNL1 KRT35 AREL1 SUN3 SEMA3F SPATA31D3 DUS1L NEK9 KDM2B CHRM3 OVOL1 NEURL1B DPEP2NB SLC25A45 HARS2 DCHS1 GNB1 TRIM46 TCF23 HLA-DOB C22orf15 TPPP ADNP UST DMWD RFFL BTD CALD1 NIPAL1 ERICH1 ST6GAL1 PMS1 EIF3E SKIL HOXA11 RPL31 HMGCS1 KDELR2 ATP2B3 POLR3F TLE4 TSTD2 TLK2 ZNF2 ST3GAL5 DHRS1 HIBADH IGFBP3 GOLGA8R GAK SFT2D2 HSF5 CRTC1 PSPC1 GRAMD2B ARHGAP24 FCSK AGBL2 ALMS1 KIF22 DCN ACTBL2 JMJD7-PLA2G4B FAM47E FHIP1A UCP3 UBAP2 STEEP1 VAMP7 AMPD2 IFNAR2 SPRED2 PLA2G12A NBPF14 BBIP1 CYB5A N4BP2L1 ELOB PITPNC1 HAUS3 GBP5 RSF1 CX3CR1 POU3F3 MON1A C2orf88 HMGXB3 TMEM269 IFRD2 SLC25A31 ANKRD13D KCNMB4 SEPTIN10 PTCHD1 FZD4 GALNS DOCK7 TMLHE STX6 MARCHF1 ABCA5 PDK3 DNAJB14 TSFM ARHGEF19 TSPAN17 ABCC8 ZSCAN20 MAP1S LYRM7 BRD3 MAJIN XKR4 CRAMP1 FBXO39 METTL17 TNPO2 IRF9 PABPC1L2B MCPH1 MINAR1 LBH SYNM BMP2K NALF1 DEPDC4 SAMD4B HEXA HNRNPU DYNLT3 SESTD1 SCAP SLC4A4 RHOBTB1 FLII ZNF486 AP2A2 CHPF SMAP2 CSNK2A1 ZBTB46 RFC5 BRAF NEMP1 PAX5 RTL8B AAK1 FBXO2 DTNA TMOD3 GMEB1 EEA1 CRB1 IL1R1 UBR4 LTBP3 DDX20 ABTB1 UTP15 ZNF697 PHKB TBC1D8B ZNF776 KMT2C ITGB5 BAIAP2 RNASE4 ARID1A TPD52L3 MBD1 ITGAL RCN2 WDR73 VWA3B MLYCD THAP9 FLVCR2 AP1S1 COL12A1 MAP3K9 ZNF280D TFAP2E OR2AJ1 VEGFB COLQ ZNF445 RYBP TTC26 HNRNPUL2-BSCL2 DRG2 RNPEPL1 CPSF2 NLRC3 GBX1 SAFB ASTL HECW1 ARL6 RTL10 ZNF519 MEF2C FAM174B PALS1 AFF3 DARS2 MYB KLF3 FZR1 DPH1 ANKRD37 SLC44A4 ESM1 ESPL1 B3GNT5 PTGER3 DBNL RPTOR ERBB4 TSPAN32 ACTR3 OR4P4 ZAP70 OR2T33 DNASE1 SLC6A2 LPP ZNF554 ORC3 HECA ATRX TVP23C TNIK GNG8 ELP4 IRS4 MACF1 AIFM1 WWC2 EPB41L4A LILRA4 HSPB8 PCMTD2 MNAT1 ZDHHC2 ADD3 ILRUN RLBP1 SLTM MALT1 TRIO MEX3B KANSL2 DENND11 ZNF385D KIF7 ZNF783 ITIH5 WEE2 IQCK NBEAL1 MTM1 GPR107 ZBED3 SMARCC2 AP1G1 ENPP4 FAM234B SMARCC1 FOXQ1 GNAI3 NAXE SPATA5L1 SLC37A3 CD4 RAD51B MMRN2 NDST1 COL20A1 ZNF248 PTPRE FAM20A PELP1 NXNL2 CLRN1 ZNF230 UGT3A1 TSPAN3 THOC5 MSN FBXW8 SH2D1A RHOF SMARCD1 DUSP22 SH3RF3 BPIFA2 ZNF347 TPP2 C19orf53 PKD1 ATAD2B ARHGAP17 GPNMB GPR83 TTF2 KCNT1 SLC39A14 NGRN RGS16 EXT2 TCIM DIAPH2 CLN8 MECP2 PNPLA4 DIP2C TOP3B NFATC4 BCO2 PDLIM7 MSH5-SAPCD1 MSL3 TIMP3 STAG2 FUBP3 HRK MOB3C LRTOMT MAML2 NFATC1 ABHD4 ARHGEF12 CCR9 RPS23 PLN LYPLA1 B3GNT9 SLC35F6 UAP1L1 SLC18A3 YOD1 RALY ADGRG3 YIPF4 PTGDR DBNDD2 DGKH ZNF581 CDK12 CCDC39 ANKRD10 TAGLN2 SLC16A7 NEDD4 ZRANB3 PPP3R2 KRT13 DCBLD2 PRKAR2A RBM33 ATP6V0B YTHDF3 SPATA17 ZBTB38 PGRMC2 KATNAL1 PAQR6 CFAP61 OSTF1 FBLIM1 FYCO1 RAPH1 CTIF CFAP57 TMIGD3 MYO6 MINK1 DNAJA4 RAP1GAP2 LRRC38 ATP11A RBM24 ZNF154 FAS KRTAP7-1 CNKSR3 BTBD17 CSTF3 PRKCI POU2AF1 R3HDM4 RASAL1 COMMD2 WIZ DDAH1 ACBD5 TMC2 SLC35B2 SPRYD4 MIER3 CRYAB LRRC58 ZNF8 TRIM72 PRY2 RTN3 DBX2 KIF11 IPO8 SKP2 SYNC LRRC75A PHC3 TNFRSF11B RNF34 OR7D4 FAM102B TMCO5A CCNI2 OPN4 ARF4 OR13G1 NKRF CCSER1 SLCO3A1 ZNF692 SH3GLB1 SECISBP2 SLC12A6 KBTBD6 HMGN3 URI1 HDGFL1 FAM133B PIK3C3 PAG1 OTULINL APOL4 KRTAP20-2 SCML2 BCL9 GPCPD1 ZBTB2 ZIM2 LETM1 GALNT5 COMMD7 USP10 B4GALT1 CNGB3 TEX261 CHD6 ESYT3 ZFAND4 CD40 FGD4 MRGPRX2 GDI1 ZMYND8 PDXK KRTAP5-9 TAP1 ZNF670 HSPB11 SPICE1 IFFO2 ZNF609 FAM135A STRIP2 GOLGA6D RSKR DEGS2 INTS8 ITGA1 EPHA5 ERMP1 GTPBP3 TSGA10 THAP8 FAM156B PACS2 ZNF721 POU4F1 BCL2L2 SGIP1 ARL4C ATG4B ZNF641 PTPN20 ADGRL2 PLEKHG2 SNU13 PRRG4 GPR22 NCEH1 AQP4 GALR1 ARMC8 BAG1 ORMDL3 DCUN1D3 RNF144A SLC7A14 PRKCD LPXN DSC2 RBMS2 GRIK4 TP53INP2 SERPINA1 ANTXR2 SMYD5 TMEM74 RET RBM19 FADS2 ENAH PDZD9 MID1 ATP2C1 CYP4F3 RASEF GABRG1 RNF103 MAMSTR AHCY TSHZ2 ZNF366 EGR1 SLC12A2 PPTC7 KIF4B STON2 GLP1R PTGFR TRIM37 GRHL2 STOX2 IREB2 CDR2 NLGN2 PSMC6 KLRC2 ABCC4 DLG3 GPR12 SSR3 NAA16 TRAT1 MFRP CST9 STRADA MAGT1 DYRK2 KLF6 SAMD7 THBD NR5A1 SRR CYP4V2 PRH1 TM2D3 SCUBE1 SMIM8 ASAP3 BHLHE22 CD180 POLA1 PROSER1 ASPH ACCS RRAS2 LTBP2 SRP68 ERN2 HECW2 PKD1L2 MAP10 PITPNA NDUFS2 PRPF4B KDM1A SLC25A22 ILK ZNF20 SEC14L3 PEAK1 PRR14L PITX2 PITHD1 DYNLL2 CCNL2 FABP3 GTF2F2 MYPN CAMKK1 PHETA2 SATB2 KCNJ9 CDADC1 ADAMTS19 AP2A1 ENDOV SMPD1 BHMT2 TH ANKS1B CDH18 CHL1 VANGL2 TBC1D2B CCDC32 SMIM33 AKAP10 FA2H STC1 ALG13 TTLL7 NAA20 EFCAB14 STOM SLC13A2 TMEM8B WWC1 PLEKHA3 PLEKHG3 FRMD6 DGKZ EFNA1 YY1AP1 ANK2 SENP6 ARPP19 OR2A5 FKBP10 ZNF516 VLDLR LARP1 DBT CGGBP1 PDGFB KLK2 SAMD8 ANO2 TLR8 HPS3 NAA50 SMIM7 MRFAP1L1 KCNK17 NGB ANGEL1 CARS2 KIF21A ABCE1 CPN1 RHBDL3 SUPT3H PWWP3B RSPO4 EDNRB SCD EGLN1 FAM167A FAM229B PHF19 GRIN2B CMC1 COX20 CACUL1 NT5DC4 SHANK1 NR1H2 ARHGEF39 CD226 TCTN1 RBM14-RBM4 UBXN10 STAT3 LIN7A SLC38A2 PPP2R5C SYNJ2 TRIM26 ST3GAL6 GID4 MPZL1 VPS9D1 LRRIQ3 PARPBP CRISPLD1 RUVBL1 ADAM9 ZSCAN29 DDX52 TMUB2 APAF1 NEK1 MPP2 TNK2 IFNGR2 ANKRD11 PCNX4 CEP83 EIF5A2 PKP4 EGFLAM TMEM238L STX7 CPM GOLGA6L2 BCAR1 ATXN3 C5orf49 SETD6 CDC42EP2 CFAP299 PLD5 P2RY2 GATD1 IER5L GPRC5A DGKB TGFBR1 MTCH1 SFMBT2 C1orf52 CDC42 TTLL2 VPS33B NAA10 LRPPRC ACVR1C DPH6 TM4SF18 SETD5 TMEM169 POMGNT1 TNKS SLC35G1 SNX2 PYCR1 TSNAX ATM TIE1 EDF1 TUB KLF12 TRMT13 KCNK1 DAPK2 EREG AXDND1 LRRC15 SUMF2 CENPA DCAF7 RNFT2 PARP2 NDFIP2 AQR CDCA7 USP45 ZNF134 ZSWIM6 PPIB CCDC127 HHIPL2 KCNH6 TMEM183A SSUH2 PYGO2 EIF3F KCNJ10 CCNJL USP53 CRYGN RCAN3 STYX LYPLA2 DOK6 LHFPL4 CINP GPR149 NMT2 SLC35B3 CENPC G0S2 PFKM SGMS1 DKC1 CCDC40 GNL1 CCDC160 TRIAP1 SCARB1 GRAMD4 GOLGA8S ATG9A VARS2 PTPA ELP3 GREM1 ITPR2 SUN2 UTP23 PRKAG2 GPAT2 OR6K3 MIGA1 PELI1 C8orf34 CELF3 SLC35C2 MLLT3 CCNT2 BCL2L13 ABLIM2 RAB7B GOSR1 BTG4 CCDC30 ZBTB39 NUP50 SHC3 C7 WASF3 DYNC2H1 GLYCTK MTUS1 ARFIP2 SRP19 UROS MFSD1 HARS1 LRP10 LEMD3 PUS7L KHDRBS2 B3GLCT SOAT1 HCN3 STX16-NPEPL1 ATG16L2 ATL3 EXOSC8 LASP1 ACHE MIEF1 PLAA OR2T11 EGR3 INSYN1 IFRD1 WDFY1 KCTD9 PPP1R3A PRNP ACTN2 ATG9B NUDT3 RAB4B-EGLN2 PALS2 RAP2B GABPA CAMK2G FGF14 POLB FBXL2 PTBP1 BRD1 KRBA2 CDC7 SNX7 MTMR9 MCCC2 CPT2 LIMS4 TRIM58 NSDHL GLRA3 NAA35 VPS8 POLR2D STAG3 MTX3 TOPORS TMEM94 KLHL32 BCL7C ARHGEF40 OR51M1 GAPT CCDC142 MVB12B FXR1 ZMYM3 ZBTB14 CASP3 DMRTC1 UBASH3A SCLY ZNF831 TPRA1 CA7 CNOT9 ENTPD5 RNF213 TPRX1 DHX9 CNEP1R1 TMEM67 NCKAP1 XPR1 PKN2 PRPF40A MAGEH1 ZNF333 AGO1 ZNF213 DTD1 INTS11 GLYATL1 WDR31 TBC1D3F SH3RF2 NEK11 MST1R CNTN3 GFRA3 RTN4 FASLG WASHC4 PRDM11 ZBTB37 RPL26 ODAD2 GOLGA8M CACNA1D PRKAA2 UHRF1BP1 CILK1 BTBD3 ARMC3 ACTL8 ACSS1 FOSL2 BBS2 FBXO21 GRB10 TBL1X PCDHA10 LANCL3 CLSTN1 EPHA3 HNF1A R3HCC1L RFWD3 RBM12 MOV10 EDIL3 INSIG2 KIF26A TSPAN15 MS4A1 DEDD MIXL1 PTPN1 LURAP1 VPS37C RCCD1 DSC3 GPATCH1 WDR11 NT5C3A LHCGR SAP130 EZH1 TMEM65 ANAPC11 VWDE PCDH1 TPRG1 MRPL32 TRIM67 PMEL CDK9 KLHL33 CDYL2 GLRX3 CAPN13 DDIT4 UVRAG DNAJA2 RANBP3L ZNF619 SERINC5 CCNL1 USP6 ADGRB3 INPP4B PCDH11Y ELK1 GMPPB BMPR2 KLF7 B3GALNT1 MYO3A AARSD1 GDAP2 PIMREG DMKN FRMD8 USP31 UBE2R2 RNFT1 HIVEP2 MYRF BEND2 CUL5 SNX27 HACD3 MTA3 SYT6 MICOS10 CHTF8 DENND10 FOXO4 NUBPL CERS5 LYG2 SOX5 KIF6 OTX2 SFT2D3 DGKI IL12RB2 RIN3 DERPC EIF5 AIFM2 UBL3 SEPSECS MED17 DIP2B ANOS1 LPA COL4A4 TPP1 PNMA8B IL17REL UTS2 PREB GJA3 TBC1D30 COL28A1 TMPRSS5 ANKFN1 HOXC12 UBE2C ZSCAN2 TFDP1 TOX4 SEC24C DENND1B LHX3 TUBGCP2 RPS15A TAF1 SLITRK5 SKOR1 BOLL PIH1D2 METTL22 TEX35 PPME1 PIK3CA LARGE1 HTT DCUN1D1 TSR2 RUSC1 MBNL1 KDM4C SLC6A5 NUP160 TPGS2 NOPCHAP1 DAZL DCUN1D4 TRIM64B ZKSCAN8 ATAD3C HNRNPDL NECTIN3 IFI44L IGFBP1 GGT7 FUT9 ACSM1 ZNF766 PTPN21 MOK TDG PLAG1 MYCBP2 MRPS5 SLC6A19 COX18 OGFOD3 ENTPD4 CCDC82 THAP7 TP53INP1 XPO1 ZNF300 LATS1 FDXACB1 ZNF777 LDAF1 CAMK2A SMC1B FXYD7 PABPC4 EPHA6 CXCL12 DDX6 PCDHB10 LHFPL1 KCTD6 CA10 AP3M1 DIAPH1 ZNF71 ZDHHC5 OR2A42 ZNF460 OR2B11 C19orf12 MRE11 COMMD10 COPS8 SACM1L ZNF784 IGLC1 ADGRL3 GADD45GIP1 FAM166C ASB6 MRPS23 EHD3 PURG PGAP3 EFEMP2 SF3B1 CAV1 GNPDA2 NMNAT3 NOB1 STRBP PIP4P2 CEBPG ATRIP RNASEL ABHD3 DNAH9 GTF3C4 HELLS TNS4 FANCI TMEM260 NAV1 PIGN RBM22 SHROOM3 RETREG3 CBWD3 ALX4 CACNA1S GCFC2 MSI2 CD300LB LRRC7 IL6ST NCAPD2 C1orf74 PRSS23 ANK1 WNT2B DCP2 WASHC2A GLYR1 PPM1L TMEM177 SPATA20 CXCL1 C1D PROB1 EPM2A USB1 EXD3 SGMS2 SFTPB MXD3 FAH MON2 TNFRSF10B USH1G FRK GABRR1 IKBKE RELA DBN1 TRIM71 WNK3 PKMYT1 TSEN34 SMIM14 SLC26A9 DUT TXNDC9 MAP2 LARP1B SPATA2L FBL C21orf91 CDC73 CFTR VSX1 CAMK1D GSTM3 ZNF280B GOLGA8F C19orf54 SPRY3 FLRT2 SLFNL1 DZIP3 CD48 NOMO2 TNRC6C KMT2A RIT1 FGD5 ARHGAP44 MED9 AFMID FBXW11 TMEM201 ETAA1 SLC12A5 MLLT10 PHF14 ATP6V1G2-DDX39B GOLGA6C S100A10 NOS1AP EIF2AK4 FAM3A DGCR6 RRP12 GARIN5B MEAF6 CLEC12A MAP9 ACBD4 VSTM5 SIRT3 SPSB3 H2AJ DPP8 KIAA1614 OTUD7B IL7R ZSWIM5 FAM83F TLR4 IFI35 SLC39A3 KIF13B BSCL2 NSMCE3 PCDH9 DIPK1C ADAMTS13 ITFG1 ACVR2B CBLN3 TTYH1 IYD C3orf18 SUFU CYB5RL ARHGEF33 HMGCLL1 SOCS2 PPP2R5D EXOC1L GLB1L3 XKR6 NKAIN2 WDR5 SPATA21 GBP3 RARG MYO7A CD96 C9orf24 ACVR1B C11orf24 RBMS3 SLC41A3 PSMD1 DSEL DNAJB5 DPEP2 IFNGR1 NOL9 SLC29A2 MATN2 TDRP COQ8B MRPL21 ANGEL2 FAM162A SAGSIN1 ARHGEF6 TGFBR2 ORAI2 INAFM1 ITGA11 PHF13 DDX60L CTU1 COBL CFAP20DC TSSK1B NFX1 MBD3 MDN1 TLE6 FOXN1 AMOTL1 IER3IP1 RASAL2 PEMT ADPRM ZNF575 TOMM40L NFKBIE KLK10 PHF20L1 ADAD2 ST3GAL3 RAB11A PLCD3 DYNLT5 TTC6 PPP2R2B CLEC18A OGA WSB2 SOWAHC ZNF431 TBC1D25 VSX2 GPD2 SCUBE2 MYCBP VANGL1 FLOT1 TOR1AIP2 PIGO KIAA1328 SLC35E3 KCNB1 CANX ZMIZ1 ENPP6 TTC23 GBA3 RNF125 MTRF1 MCM6 CIRBP SEC14L4 FRZB SHROOM2 RPGR CES5A CATSPER2 USP22 STARD9 CLDN8 TMX2 LYSMD1 GPRIN3 TSR1 SEPTIN8 ZFHX3 TMEM126B PCSK5 CCDC85A FAM107A HEMK1 NDUFA9 RSRC2 TBC1D2 EGLN3 FZD6 HS6ST3 FBXO45 SLC25A12 CUX1 GPX3 TRIM64 GRM4 TBCK TCP11L2 KLK1 PLB1 RNF130 CPSF4 SMIM15 MAP2K1 KCNMB2 SLFN5 ABHD14A-ACY1 ST13 ZMAT4 MARCHF8 TECRL UBR2 CCL15 GP5 MAK MORC2 SAE1 GOLGA8T SGCE PRSS8 RASL11A TCF7 CTAGE1 SAMD9 DTWD1 STRN4 S100A7A SETX YKT6 SPARC WWP2 KCTD12 CTSH UBE2V2 PPP2R5E COL5A1 FBXL18 IFT57 IL20RB ZNF592 TNFSF9 KCTD21 CFP DCTD CNIH3 ZNF37A FICD STX16 PAQR9 ALDH3B1 KIF21B DLAT KCNJ5 CDC5L LMNA TBCD KPNB1 FOXO3 LARS2 TRIM59 SRSF5 BRD3OS PPP1R18 DCTN2 PANK4 HIP1 RAET1E DNAJC24 WNK1 CDKN2A AHNAK2 CARMIL2 OLA1 RFX7 TTLL10 ADGRV1 NKX3-2 CSRNP2 RASGRF2 TRPS1 SSBP1 KPNA6 SUPT7L CASP2 HS3ST4 TESC DOK7 YTHDC1 PDHB ERN1 AGMAT NOTCH3 ADAMTS5 RFLNB QTRT2 NF2 ZKSCAN7 VTA1 PRRT2 PCDHGA6 CA6 CLPP CEP57 MTMR3 TXNDC17 TRIM25 DDX3X CRKL GATM RASA3 TENT5A EPB41L5 SERPINB12 ITPRIP SH2D5 DHX36 TIGD5 NUDCD1 HDC KLHL3 TFF3 CHCHD4 TNR APPL1 HEYL RAB43 ADCY9 DPP10 STUM ZNF816 PNPO SFRP1 MAP3K19 TDP2 DAAM1 GOLT1B NEK3 SSTR4 UPK3BL1 TTC38 CD40LG CES1 TRAF3IP1 SMARCE1 STK17B B3GAT3 ADD2 PKD2L2 URM1 TMEM106B PISD LRIG2 CLIC6 SKA2 USP1 SAR1B TTC7B SCN9A DPYSL3 INSYN2A RBL1 PAK2 SLC51A GLS2 TEN1-CDK3 MNX1 ATF7IP2 SPRING1 MS4A14 C6 WASF2 SLC16A10 IGF2BP1 KBTBD7 MDM4 MAMLD1 ATRN SNCB PUM1 OR4F6 NDUFB9 CCDC88A CNOT6L PMS2 AMZ1 RNLS KCNA7 ZNHIT6 SPEF2 DCAF8 TP53TG5 CDCA8 APBA1 ACTN4 RAB2A HPSE2 DLG1 EIF2B5 KCNJ2 VPS33A PIGX GRAP2 POLR2G CNTNAP1 PSTPIP1 APOL2 CNBP PDHA1 RTKN2 BYSL COX7A2L ANKRD36B PMPCA KLHL2 U2SURP TMEM151A CUBN HERPUD2 SLC7A6 CSPG4 IGF1 G3BP1 HSD17B2 FAM214A UBE2G1 ADCY7 SSC4D FRAS1 PELI2 DCAF5 B9D1 NKX2-5 LILRB2 TPRG1L CSTF1 NEPRO DHX35 IMP4 SLC33A1 DCLK3 ZNF131 ADCK1 ANKRD13B USP47 TRIM33 G2E3 IZUMO4 NWD1 PPP1R13B TOP3A AATK SLC16A14 UBE2F-SCLY NDUFAB1 SLC4A8 BAK1 CHRM5 COX6C AP3S2 FXYD6 MS4A10 TTC37 TET3 GOT1 ZNF576 NUDT17 UNC5A TMEM39A CASTOR2 SLC13A1 PIKFYVE PI4KB MTMR1 SYNGAP1 B3GAT1 BET1L FADS1 CXADR TSC22D1 DKK2 RHOT1 EPS8 TANGO2 GPR153 ARHGAP21 SAAL1 HERC2 RIOK1 CACTIN USE1 HOOK3 HNMT GINS1 ITM2B BSN CAND1 PAPSS2 CLCC1 NASP ACSL3 CDC14A CDK11A COL9A2 RWDD2A IMPDH2 ENOX2 COPS9 INTS6 TMEM240 A1CF GAREM2 GPR37L1 OGDH ASB13 GSS ZFP14 CECR2 DCP1A KCNA1 XNDC1N CCN4 C10orf67 BIRC5 PPP3CA UTS2B SLC1A7 CES2 DDB2 CPLX3 TMEM250 GOLGA3 NKD1 MTRR BBS7 GLE1 GTF2H2 EXOSC2 MYL12A BHMT LAIR1 NPC2 AVPR1B GTF3C3 GUCY1A1 HS3ST1 IL17RC RBPJ METTL2B ZNF280C PF4 MSH6 DAPK3 IPO4 SRSF6 TRIM41 MRAP KATNIP SLC6A15 FRMD5 C1orf216 FRRS1 SLC38A10 IQCM ULK4 TRIM2 RNF217 ATAD1 UBR3 DNM1 PLEKHG7 HYOU1 NOP9 ZNF25 ATOH8 RXYLT1 ASCL5 LLGL2 USP7 FAM98B OR2T1 OTUD3 SPPL3 HNRNPR ENOSF1 MEIS2 PCDH7 ZNF75A CDH10 KL SCP2 ALCAM ABHD2 MAFK ORAI3 SON MPP4 NUP37 CDS1 ERLIN2 SLC22A6 OTUD6B TVP23A TMEM117 ADAM19 TSPYL4 CELF1 BARX2 DAO ADAMTS16 TASP1 TMEM202 EDNRA NIPBL NBPF19 EFHC1 SERF1A ZZEF1 ZNF24 MICU1 OR2C3 ATPSCKMT PIP4K2B HHLA2 PIK3AP1 LTBP1 PRSS21 RYR1 FGF8 NDUFA2 LRRK2 APPBP2 NKAIN3 CPEB1 INO80D KDM5A CNTFR EPX ZNF728 ABI2 IKZF3 PRXL2C SLC38A4 ATF1 MYOCOS CLSTN2 ARIH1 LGI4 RPL32 CFAP298 CDC6 PLD1 WDR6 SULT1C2 CHI3L1 WDR62 ITFG2 MOB4 PKHD1 ARL2BP HPS4 KIF2A TBC1D3E NOMO1 OCRL PABIR2 BOD1L2 NEBL DIDO1 RARB ZNF320 CCDC18 PLA2R1 CBFA2T2 CNOT6 STK40 PIGP PRRG1 HLF ADAMTS2 CBARP PJA2 PKIB TCF4 GABRQ MED12 C1orf141 FXN PHF1 AURKA SRD5A2 TBC1D9B GPRC5C AP3B1 FUBP1 IGLL1 IL5RA MLN TIAM1 PERP FBXO36 MTR GOLGA8N FAM126B ASH1L VAMP4 PHACTR1 RDH8 TTC21B IQSEC2 PLXNB3 EML5 UBQLN2 TAB1 C5orf47 HNRNPUL2 ACVR2A MEGF10 NR1I3 YWHAZ PRRX1 ANKRD52 KLHL9 GAA ARHGEF16 SCN1A RPS6KA6 STRC GDF9 PFKL BCL2L15 SNN AURKB OR5K1 ZC3H6 PRPF39 DISC1 GABBR1 DZANK1 PROX1 CLTCL1 LANCL2 SCN3A ALG6 LAMB3 MOGS CATSPER4 FMN1 CARD14 SEPTIN7 ASXL2 ZNF891 RALGPS1 ARHGAP42 CCDC6 NCOA3 SPCS3 RASSF4 CHAF1A MTHFD1 SLC17A9 TPT1 ZFPM2 SEC63 PIEZO2 KCTD10 SLC35F1 TOR1AIP1 SYTL4 VSTM4 G6PC2 MUC5B CTSB ZFP36 TMEM170A PXN CCDC77 RAD1 CYHR1 SIRT2 FANCA PHYKPL VTI1A GANAB GDF2 ASAP1 PCBP3 GVQW3 HNRNPA0 GRM6 PGAM1 ATP8B4 MEIS1 BMP8A SPOUT1 ZGRF1 COL23A1 NSD1 GLDN NEDD4L WDR91 NHLH2 NCAPD3 HDGFL3 PDGFD CTAGE9 ERCC1 AGO2 TRIL TMEM79 CREBBP GTF3C5 ITGB1 BVES CCL14 PAX1 AP4M1 KCNG4 TUBA1A ABCC2 RIMBP2 DNAJC16 TTC12 CNGA2 SNX13 CSPP1 NAIP CHST10 GMCL2 RABGEF1 MAP1LC3B THY1 P2RY8 CYB5R4 DCLK2 LRBA MEMO1 CACFD1 NAPB FNTB KRT8 TMIGD2 EPHA7 LMAN2L DSE OSBPL3 PPOX HAND2 SERTAD1 MARVELD3 TMPRSS13 CREB3 DGAT1 NKIRAS1 KRTAP1-5 RAD54B LRR1 GPATCH11 BARD1 IGFLR1 CASP14 FDX1 GRIA1 C19orf47 IL13RA1 EIF2B2 RGS8 WNT5A GNS C1QTNF7 DMRTC1B UBE2D3 LRP5 SPIDR SLC25A34 NEFL ANKHD1-EIF4EBP3 COX7B WNT9A TRAPPC5 DND1 ATXN7 IL21R STK3 REPS2 TGIF2 PRDM6 RNF150 CYP2C9 ORMDL1 WNK4 COL19A1 GFUS ZNF468 C1GALT1 BAG3 NKAP THAP5 CA5A RIC8A RUNX1T1 USP35 PIK3C2G HNF4G ZCCHC10 NOL11 PDCD6IP PSMD2 ZNF740 AP2B1 ATXN2 RS1 LAMC3 PCDH15 ESR1 SYT7 GCNT2 PGGT1B SURF4 PLEKHO2 KCNN3 GLOD4 BCAS2 SGCZ SPATA9 OLIG3 FAM186B CHMP3 EDA2R DNA2 PLCXD3 RHOV CLN6 ANAPC1 PIM3 DMXL1 PTPN14 APTX SNRPG DMTF1 BMS1 HTR5A GRM1 ACOT9 PDE4D BPIFB6 CNTN5 ZNF514 KCNMB3 WDCP ZNF705A PDE3A PCDH11X OR10A6 TMC6 DMGDH RASGEF1A NTPCR TRANK1 C10orf143 OTOF RIMS1 GXYLT2 RCN1 HIPK3 RRH MDH2 DRP2 TXNL4A FCRLB FGFR4 PDZD7 LIMS1 SREBF2 KIF9 MFAP3L PIK3CD DERL2 SHISA8 UXT ZXDC ALG11 APEX1 CEP68 VPS53 PSEN2 SLC5A3 TBC1D3L CHST2 SPATA2 FZD10 NAA15 ASB1 FRMD4A SAR1A MCTP2 LCMT2 PTGIR TMEFF2 LMX1A NLGN3 PRM1 MEP1A DAG1 TMEM121B GP2 CHIT1 UCK1 TCAF2C PYROXD1 TSPAN2 MRPL9 RUNDC1 SDHAF1 ARID2 PPP1R3F FAM91A1 GYG2 DYNC2I1 LINC00672 ZDBF2 CTSF ZBTB4 HUNK TSPAN14 CHD1L NAA40 NUDT16 CNKSR2 BEND4 GFRA2 ZC2HC1A ZNF74 TR SNX29 WWP1 FILIP1 SAMD5 STAT1 TMEM62 COL8A1 IGFBP5 RBMY1D POLD4 MRFAP1 SSBP2 ANAPC10 LRRC36 CDCA5 LIX1L IRF2BPL REEP1 SLC2A1 CREM LSAMP RPH3A CWC25 ATP2A3 PTGER4 CCR7 KCNQ1 PROCA1 WEE1 NR2F2 ANGPTL1 CXCL17 CCDC47 PARP8 ZNF215 FGD2 SLC26A6 DTX4 EFEMP1 TIMM8A BNC1 P2RY14 SRRM1 AJAP1 ALS2 COMMD8 CYTH4 NAP1L1 PTDSS1 SLC38A6 FKBP8 GALNT15 GLCCI1 KIAA1109 POT1 MMP11 C3orf62 MYO1E BEST3 ZFX ABCC9 SNAP29 HMGCR CAPN8 SAXO1 ZNF727 IFT74 CNTROB CARHSP1 PLPP4 GREM2 CCDC102B HERC1 IQGAP3 CRADD INPP5K NCOA5 CCDC174 BLNK VASH2 SLC25A40 IL19 IRAK1BP1 COPB2 CTR9 OR8G1 UBR1 CDC27 COL9A1 VPS35 GPC4 VAC14 TMEM178B PSMD9 SIKE1 COL4A2 HAS3 KDM5B LUM UBASH3B GFPT1 HMOX1 ARHGAP32 GGACT KPNA4 FAM126A MRPS10 TRPM7 FEM1C DYRK3 PFAS TMED3 PPP4R3C ADGB ZNF528 U2AF2 MKNK2 SMAD2 CPLANE1 ZNF44 EFNA5 YWHAG CCS NOL4 TMEM68 NXN PPIH ZNF555 EIF5B ZIC5 UTP25 LIMS3 NIBAN2 CNTLN ASL METTL14 GATA3 SLC2A14 IL17RD PARP12 TEX13C ALG10B KLHDC7A ARX PPP3CB SRSF7 LSM14B MPZ NAMPT HECTD1 PCDHGB7 VKORC1L1 CYP4F22 MAP3K21 ZNF493 WBP2NL TLN2 TBCEL ELOA HDAC4 RPS6 HS3ST3B1 STK36 FAM102A SFXN1 TRPC5 CTNNBIP1 TEP1 YAF2 KIDINS220 ACP5 TBCE AMFR LIAS MAPK1 ZNF705D FNIP2 PGF HSD17B3 SAG G3BP2 PYGB TSC1 DKK3 OARD1 DDX31 TMEM70 NHLRC2 ZNF430 AASS POSTN JAML ZNF793 CYP27B1 FTO CALCB NLRP2B RBM45 USP46 CCNJ PDCL DNAJC5G LILRB3 DCAF6 DHX8 ZC3HC1 CT55 RBM14 SF3A1 IQCE CD99L2 LVRN RPGRIP1L SPOP LRRC23 PTPRF DAZAP2 RASL12 IKBKB PPFIA2 BOK TYK2 NRL GPAT4 PIP4K2A TCL1A MEX3A ZNF268 CMC2 MYLK UNC5C FCHO1 HOXD10 OR2L2 TRIM66 AKAP6 SRPX2 CFAP69 RINL CTLA4 MTMR4 CIB3 SNX9 MINDY4 CBWD6 ST6GALNAC4 KRTAP5-8 UQCC3 LCN10 SAMD11 TSC22D2 RELCH ARHGEF15 CLCN1 RAB3GAP2 RIOK2 CEACAM21 SLC22A15 ZNF142 CCPG1 DCBLD1 TRDMT1 PDGFA P3H2 NAPSA CFL1 SLC38A3 EML6 PIGQ CRACD BRD7 GBP1 LINGO2 OPCML AKAP12 BLCAP CEMIP HLA-DPB1 SLC25A27 KIFBP SLAIN2 AMMECR1L LRRC37B MGAT4C RHOXF2 CTSE CEP85L ATF2 SLC39A13 SLC35A2 FBXL22 CCNT1 OR52A5 HELB LIPG PHACTR2 FEZ1 SLC35E1 CTPS1 APOD LAD1 EHD4 KCTD11 DENND6A NCKAP1L NOX5 SULT1A3 MAPK8 SYNCRIP ESS2 WNT11 FAM47E-STBD1 OSBPL1A PLCZ1 STARD4 ZNF589 MED13 NLRP11 SIGLEC12 CHRNB3 SCN1B RNASEH2B ESCO1 JADE2 KLB RFC1 ADORA3 COL6A5 ZFYVE28 TRNT1 NSUN6 ANKRD44 BMP1 CYP3A43 DNAJC21 PDE7A AKR1B1 FOXS1 GRID2 XPNPEP3 MTDH SHB CSKMT LMBR1L MYCN RSBN1 TBX18 PCDHGA8 RPS6KL1 RALGPS2 NTF3 TRPC6 DPP9 FER C2CD2L SLC43A2 EXTL3 LIG1 EHMT1 TMEM97 SUZ12 EXTL2 CCL1 MACC1 FAM72A MCMDC2 CDK5RAP3 GGCX TCEA1 ZNF853 SELENOI SPA17 SUSD4 KBTBD4 ARSD RB1 CNIH4 IFNL4 FKBP15 MPV17 RNF24 GPX8 REPS1 TENT5B ALG8 ST3GAL4 TBC1D3D SH2D1B NTNG1 SSR1 RBFOX2 SETD9 NUP107 MYOZ3 PDE4B ADAM18 DDX10 TMED10 CASTOR1 LRRC8B MAPRE2 RAB11FIP1 CSNK1G1 ZNF26 ZNF169 KIF13A RAC3 TCTE1 PCDH17 PHF8 CLIP4 CDK11B LUC7L3 RAD51AP1 ARL2-SNX15 RC3H2 SGCD TMEM87A PHTF1 CMTM5 PKIA GFER SEC62 NRIP2 RIC8B SBDS CFAP107 TAF3 GIPC3 MAN1B1 P4HA1 GOSR2 SP3 UBE2Z LRRC20 LCP2 GAB3 COL4A1 NR2C1 SEZ6L ADAMTS3 OSBPL2 STK19 TBC1D7 TNFRSF13B PCYOX1L VPS54 SLC39A8 GLS STS PCDHB12 FFAR2 DCAF10 LHFPL5 PRMT9 GLI2 ALDH1A3 CLSPN MAP1LC3A PLGLB1 ENTPD1 OTUB1 GINM1 STK11 PLPP3 ANKRD13A PAXBP1 FIZ1 EME1 DEFB112 NLRP1 UNC80 BUD13 FNTA RPS27L TGFBR3 TATDN3 CTAG1B MCUR1 NUDT4B CASP9 MAP3K7 PDCD1 ZNF512B AKR1C1 MIPOL1 SPSB4 PPIP5K2 OR51D1 PALD1 BRD2 KLHL34 CHURC1-FNTB LAMP2 FAM53B RPS6KA1 ICE2 ZNF746 SLC7A1 MARS1 ATXN1 PRKCQ KDM1B SSPN IGSF9B CNOT8 KIRREL3 APH1B USP36 ADAM17 UBE2D4 CPNE1 ZC3H4 G6PC1 SEMA4F FKBP1C RPL37A ALG5 NCOA2 FAM118B CNDP1 S100PBP ABCA2 SLC35F2 ATP10D MESD CTAGE8 LCP1 IGBP1 PCDHGB6 TRAPPC14 FAM210B RASSF8 TRAK1 STXBP1 AGPAT5 TBC1D3K DOC2B GHRHR ABCB6 HINFP MRPS25 SERF2 RABGAP1L EIF4H CD47 ACP6 C9orf40 RPL11 GRAP RORC ASXL1 DENND2B IFIH1 AZI2 XRCC5 RAB1A LRRC75B CFAP119 SHISA7 NUDT4 PCTP VRK3 SIRT1 UBALD2 CD164 BORCS7-ASMT ADGRA1 H3-3B PCBP2 MUC13 TMEM167B HAX1 NSG1 PLEKHG1 GABBR2 WRAP53 CDKL5 SMIM32 ELP2 WIPF1 SNX15 IGF2R INPP4A RNF8 BRS3 ATP8A2 ATP8B3 HOXC8 RBM15B NHSL2 LARP4 ZMPSTE24 DDX39B SLC24A3 CLTRN FAM228A SNRNP200 DDX25 TOP2B NCAN SOBP DNM1L ORC4 RNF114 ZNF207 MPRIP DNAL1 TAF15 MED19 PFKFB4 GRIA4 LRRC57 SREBF1 IDE NOD2 IL21 ARMC5 PRELP UBTF GYG1 POLD3 RAB18 CD300LD TLCD5 CYP8B1 HSDL2 LPIN1 CLTB MKRN1 PATE4 TMEM184C POU2F1 CNNM2 APC PMP22 CCDC152 ZNF233 GOLGA8G GPR139 GPX1 P2RX7 RSL1D1 SBNO1 ANKMY2 PSAPL1 RBM25 DHX58 PLEKHA5 MECR LRAT ZBTB25 SGK3 SPTBN4 CCER1 FBXO28 CLEC10A EPHA8 C8orf74 TMTC1 ETV1 MOCS2 USP28 ADAM11 NRK NAT1 KICS2 TRAFD1 CTTNBP2NL MTCH2 SPTSSB AUTS2 BACH2 OBP2B TCN2 FSD1L EPHB2 PPARGC1B CENPT CALM1 B4GALNT2 RNF123 KDSR CYFIP2 PLAUR KRBOX5 BMP5 MAP3K11 ESR2 NPIPB12 ZKSCAN2 EPM2AIP1 PZP LPCAT1 SIVA1 ANKS3 CLEC18C APRT MSANTD4 RALBP1 SIK1 GIGYF1 HGS POLA2 ZC3H12B OTOG ERCC6L2 ITIH4 EDEM3 RUFY2 TCOF1 SF3B3 SLC17A5 HGF C4orf46 SOX18 SERPINA10 SYNGR1 UPK3BL2 REEP2 HSPA12A CAPZB PDE4A ERC1 SATB1 NKIRAS2 SMIM13 YPEL3 VPS45 TP53BP2 SOX2 GINS2 AMER1 PDLIM5 SPHK2 NXPH2 BRWD1 ZFAT EFCAB11 PCDHA6 RUBCNL SLC6A3 AGO3 PHF23 SLC19A2 PVR ZNF780A DUSP13 FANCG BCL2L14 ADGRE1 MYADM SLFN12L CCDC13 ZNF652 UCK2 L3MBTL1 MOB3B SCN3B PGK1 WLS CDYL ERVK3-1 NAT14 ANKS1A COBLL1 CLPB DONSON RYR3 SLC30A9 VAMP2 PRCP XPNPEP2 FIBP SLFN14 SPIB DHCR24 DIO3 MEF2D CNPY2 NBN PRTG RCBTB1 FGL2 ZSCAN18 CEP70 ADAMTS18 FAN1 DCHS2 SLC17A1 CYBA SOS1 PLAU JPT1 KMT2E EHF PTEN FRMD3 ANXA1 AFF1 SUSD5 TC2N SETD2 SENP1 MATR3 S100P PWWP3A UGGT1 ATXN7L2 AASDH SERPINB5 TFDP3 ZFP28 RFX5 RDH13 SPTLC1 HMGB3 TOPBP1 ZBTB34 TMOD4 KCNAB3 SMDT1 SYNE2 GPR180 ZNF843 VCPIP1 KLHDC4 LDHD TUG1 GOLM1 DPYSL2 ARL1 KLC4 SYNPO2 CDK5RAP2 GK ALPK3 FLVCR1 PROM2 DUSP7 PPM1J NANOG TRIM22 ACAD8 CDH13 LYRM2 UBE2E3 MDGA2 RAB8B RC3H1 HES5 SAMD3 CCDC50 METTL8 MAB21L3 NSD2 NXPH3 SMPD4 AIDA FARSB H3C10 TGM2 STAM ZNF202 CLDN4 LYRM1 GRM8 CGAS ARHGEF7 RNF141 TBK1 PKD2 ARHGAP30 LIN54 TMEM86A RMDN2 LYSMD4 ZNF704 SQSTM1 CCL4L2 EDDM13 SARS2 BIN3 SAMD12 NMRK1 SNRNP70 ACAT1 MAN2A2 MTHFR RPGRIP1 AARS1 PTOV1 GABPB2 DACH1 CSGALNACT2 KCND1 ZACN PTPN11 CMIP ATF3 TRDN CALML4 KRTAP4-3 GASK1A PLK1 SLC2A13 FBN2 ZNF397 NDUFA11 DAB1 KIAA1958 JADE3 PHF3 DNAAF3 BTG1 DPP4 DICER1 KLHL23 NPPB ZNF148 IKZF1 SMIM22 PSMC2 APEH SLC26A8 OR2J2 ANKRD63 CAPZA2 SNTG1 TEX19 LPGAT1 BRMS1 MAPKAP1 SULT1A4 CHST1 THOC2 ALK MFAP3 CACHD1 GAN SOCS6 EVC2 SLC25A32 LMLN GON4L FRRS1L ATP1B1 GREB1 AFG3L2 SWAP70 ADAT2 PM20D2 REEP3 SYS1-DBNDD2 MARK1 MRGBP ADIG CDKN1B GRIPAP1 LDLRAD4 SEMA3A BTRC NDUFS8 EIF4EBP3 TFCP2L1 IQCH STEAP3 TRIM3 ABCA1 PAK6 RBM4 CSF1 SEMA3E PQBP1 BCLAF3 BPNT2 SLC8A3 DTX3L GMCL1 MGAT5 PLA2G4E MUC1 ARID3A MYO5B FEM1B C3AR1 ACOX1 CADM2 MTHFSD EFNA4 USP24 KIF5B MRPS17 NUP153 CSDE1 TTYH3 HSD17B4 SEC31B CIAO3 ANKAR PCGF6 CFAP97D2 COPZ2 RNF180 DCLK1 GPI ASF1B RNF215 PACRGL TAF2 DHRSX TSEN15 TMEM237 ZFP69B CCHCR1 TRMT10B RPS3 COL21A1 NTNG2 RAI14 FAM135B E2F1 NR0B1 MEAK7 RASGRP2 TSC22D3 HAUS6 KIF16B CASP8 APOB PRKD3 RAB11FIP2 NRIP1 BNIP2 ZNF707 NRP2 TBC1D22B CDH2 PTPRB MROH7 LAG3 MOG MARCHF6 ERAP1 ATP7A CCNG2 ITSN2 CDX2 NECAP2 CAVIN1 PPARG TMEM44 DSG2 APLNR PPP2R1B JAG1 IRF6 PRDM4 CACNA1B MMAB AKNAD1 RHOA TRH EVI5 DCTN3 CIAO2B VPS41 IFT80 CXCL11 MPV17L DTX1 TNNC1 AKR1C2 IL31RA PPIG NAGA ASB3 CASD1 NIBAN1 HSPA4L SMAGP TTBK2 PLXNC1 STBD1 CTAG2 SMIM10L1 SCRG1 ZNF441 CXCL8 RPL22L1 MGAT5B FBXW2 ZDHHC24 DNAJC3 ZNF410 APLP1 ABHD15 METTL15 RAB21 DLX4 PRR33 CRY1 FOXJ2 KIAA1549L PBRM1 SCAND1 FBXO33 UHMK1 OR56A4 TADA3 ZCCHC24 ELOVL7 FGF19 TAMM41 GOLGA1 ZBTB43 VAPA TIGD1 MEA1 FAM169A S100G STAP1 NEK7 MAP2K6 BORCS5 FSTL4 INTS15 PFN2 AGO4 TCEA2 COG2 PAXIP1 ZC3HAV1 YY1 TRAPPC2L ARHGAP28 JMJD7 FAM181B MTOR FAM222B LRRC66 COX8C ANAPC7 IARS1 C17orf75 GARIN1A TENM1 NBPF1 DELE1 BNIP3 STK38L TXNRD2 FES VWA5B1 CCDC120 RALGAPA2 SLC27A1 NME6 OR6C74 STAC2 OAS3 SLC9A7 CDK6 HMX2 NPRL3 P2RY1 VAPB CSK LIMA1 C2CD4C TUFT1 CTSS KLLN ZXDA LILRA2 FBLN1 NEMP2 TMTC3 TLL2 ADGRL4 LCK LUC7L MARVELD1 ZNF76 RAF1 INF2 ANO1 MRPS33 ZIC1 SGSM1 LYST AKIP1 ARL10 INKA2 KIF20B ZNF587B NCR3 ST8SIA1 ANKRD46 SMIM12 SNX4 CCR2 LRATD1 SH3KBP1 LMBRD2 SELENOW RIMS3 DRD2 CEACAM4 UNC13D SIRPB1 PPP1R3B SDC4 TMC8 AVIL FHL2 SUGT1 HIGD1A GOLGA8A NUDT6 PHOX2A DES RNF112 ITGAV FGFR2 BZW1 CLDND1 MAGIX XRCC3 GDAP1 BEST1 PPP2CB PTK7 REV3L DDX60 RBM20 PRKAR1A ZRANB1 DOLPP1 ATG5 ZGLP1 TNNI2 CD7 RAD52 ASIC1 SGTB HR CHAT MYCT1 KYNU STARD3NL ZFP30 ISY1-RAB43 TTC39C ZNF234 MYLK4 BEND7 CLVS2 ADAL TBXAS1 DNAJA1 HSF4 KXD1 CWC27 MAP6D1 ZC2HC1C LRRN4 NICN1 NUDT14 KIF5A ASCC2 GFM1 PSENEN SLC44A1 SPRED1 KBTBD11 FAM168B DMRTA1 AP5M1 PLEKHA7 RAD51C IRAK3 CNNM4 CFC1 MYO5A SERF1B ZC3HAV1L ADAM10 REV1 GNA15 GALC CCSAP GNG4 CTRB2 CNTNAP4 MRPS22 STING1 TBKBP1 DRAM1 TSPAN33 PPP2R2C RSAD1 ZNF217 LRRC34 PNPLA1 ZNF286A-TBC1D26 AGPAT4 KANSL1 NOX4 VWF TPM1 LINGO1 DHTKD1 CRIM1 ZNF101 SH3GLB2 FLNB ACO1 NRG2 OPRL1 SCAI TULP4 THSD8 CRTC2 HTRA3 TECPR2 PAPPA2 PLEKHA1 TAF1L LAT2 SUGP1 NR6A1 SLCO1A2 HNRNPUL1 SNUPN PFKFB3 PKNOX1 ECSIT DIRAS2 AGTR1 NSD3 ABCB5 MAP3K10 RNF168 MTERF4 CRYBG3 USP2 LRRC14B RNF115 KIAA2026 MAN2A1 OBSCN SPECC1 CBX8 STRAP SLC52A3 ESD CYYR1 CCDC179 IL1RAP MTMR10 VDAC1 CSNK1D CDHR3 NDOR1 PPIA TRIB1 ERLEC1 PTPRT PER2 RGR DSG1 TRIM9 PMFBP1 RETREG2 GABPB1 DEF6 SIT1 WRAP73 RPH3AL HPGD CTSD DNAJC12 DNAJC18 NAP1L2 PHYHIP SYT13 KRT222 LPIN3 DLEC1 NMNAT2 GPR158 ABCC12 WIF1 SOCS3 NELL2 ADCYAP1 NAP1L5 UNC13A SH3GL2 CLBA1 SLC30A3 ARPP21 FAT2 VSNL1 NUPR1 MYO10 CNR1 MS4A6A SV2B ABCC3 GABRB2 SVOP PABPC1L2A RNF165 ENPP5 NAT16 AZGP1 CBLN4 SYNPR GLIPR1 ENC1 GAD1 TNFRSF10D ENTPD3 SLC10A4 RSPO2 CTAG1A GALNT17 RASAL3 GAD2 GAP43 GNG2 CD86 BNIPL RGS1 GNG3 OLFM3 IL18R1 SYT16 LRTM2 TMEM130 WDR54 CREG2 BDNF FBXO40 RAB27B SNAP25 RGS7 HS6ST2 VSIG4 CCKBR ERICH3 PEX6 ANGPT2 DACH2 SLCO4A1 PPEF1 FGF9 NECAB1 PCDH8 PTCHD4 SERTM1 PTPN3 KDM5D CHN1 TGFBI CISH |
