# Supplementary material for: miRNA-associated gene networks reveal potential candidate markers for Alzheimer’s disease
Source: Front Mol Biosci. 2026 Mar 6;12:1699404. doi: 10.3389/fmolb.2025.1699404 (PMC13002409; doi:10.3389/fmolb.2025.1699404)
Supplement: Supplementary file 1 [file Supplementaryfile1.zip › Supplementary Tables/Supplementary Table 7.docx]

**Supplementary Table 7**

**Intersecting Genes Between miRNA Target Genes and DEGs from GSE122063/GSE18309 Datasets**

| NAP1L2 RTN1 GDA MET PHYHIP SYT13 CDH8 KRT222 LPIN3 DLEC1 NMNAT2 KCNJ6 GPR158 NECAB2 ABCC12 GABRE WIF1 SOCS3 NELL2 ADCYAP1 MYT1L NAP1L5 UNC13A KCNC2 SH3GL2 CLBA1 SLC30A3 MAFF CALB1 ARPP21 FAT2 VSNL1 NUPR1 MYO10 SNX31 CNR1 MS4A6A SLC32A1 SV2B PLA1A ABCC3 GABRB2 SVOP PABPC1L2A MAP7D2 RNF165 ENPP5 NAT16 AZGP1 HPRT1 CNTNAP2 CBLN4 DYNC1I1 SYNPR GLIPR1 GAS7 ENC1 DOCK3 GAD1 TNFRSF10D HAPLN1 ENTPD3 SLC10A4 RSPO2 MLIP CTAG1A GALNT17 RASAL3 GAD2 GAP43 GNG2 ZBED6 CD86 BNIPL GABRA1 RGS1 GNG3 CAMK4 OLFM3 IL18R1 CTXN3 SYT16 LRTM2 TMEM130 MID1IP1 WDR54 CREG2 BDNF SCIN FBXO40 RAB27B SNAP25 RGS7 HS6ST2 VSIG4 CCKBR ERICH3 PEX6 ANGPT2 DACH2 SLCO4A1 LYRM9 PPEF1 FGF9 NECAB1 PCDH8 PTCHD4 SERTM1 CEP41 PTPN3 KDM5D DAW1 CHN1 TGFBI CISH MAL2 |
| --- |
