# Supplementary material for: miRNA-associated gene networks reveal potential candidate markers for Alzheimer’s disease
Source: Front Mol Biosci. 2026 Mar 6;12:1699404. doi: 10.3389/fmolb.2025.1699404 (PMC13002409; doi:10.3389/fmolb.2025.1699404)
Supplement: Supplementary file 1 [file Supplementaryfile1.zip › Supplementary Tables/Supplementary Table 1.doc]

Supplementary Table 1: GENE Primer

| Gene | Primer 5’-3’ | Accession number | Product size | Temperature | amplification regions |
| --- | --- | --- | --- | --- | --- |
| BDNF | Probe:TCTGGAGAGCGTGAATGGG  Forward:GCCCAAGGCAGGTTCAAGA  Reverse:AACTTTCTGGTCCTCATCCAACA | NM_001143812.2 | 88 bp | 59 | Exon 4-5 |
| SLC32A1 | Probe:TGGGAGGCAGGCTGGAA  Forward:CATGTTCGTGCTGGGCCTAC  Reverse:GTGTAGCAGCACACAACGGC | NM_080552.3 | 93 bp | 61 | Exon 3-4 |
| GAD1 | Probe:AGCGGACCCCAATACCACTAA  Forward:TCGTCCGCAACCTCCTCG  Reverse:GCTGTTGGTCCTTTGCAAGAAG | NM_000817.3 | 147 bp | 60 | Exon 8-9 |
| GAD2 | Probe:TGATCTATCGAAACTTTTCACCACAT  Forward:GCGTTTCTGCAAGATGTTATGAA  Reverse:GCTCATTAGGATAATGGAAATCAATCA | NM_001134366.2 | 97 bp | 57 | Within single exon |
| SYNPR | Probe:ACATTTTCTTCCAGAACAAATACCG  Forward:TTCATTGTCACTGTAGTCTTTTCGTT  Reverse:CCGTTGCAACTTTGACGTCAG | NM_001130003.2 | 88 bp | 58 | Exon 2-3 |
| SNAP25 | Probe:ATGCCGCTCACCTGCTCTA  Forward:CCCGAGAAAATGAAATGGATGA  Reverse:GGCCATGTGACGGAGGTTC | NM_003081.5 | 71 bp | 56 | Exon 3-4 |
| CALB1 | Probe:CTCATCACAGCCTCACAGTTTTTC  Forward:CTGTACAATGGCAGAATCCCAC  Reverse:GTCAGCGTCGAAATGGAGC | NM_004929.4 | 85 bp | 59 | Exon 4-5 |
| GABRA1 | Probe:AAGTAGCCAATCTTTCTCTTCAAGTG  Forward:GAGAATATGTTGTTATGACCACTCATTT  Reverse:ATTATGCATGGCAGGTATGTTTGA | NM_000806.5 | 85 bp | 58 | Exon 5-6 |
| GABRB2 | Probe:CAGGATAAAGTAGCCAATGTTTCTCTT  Forward:TCCTATCCCAGGTTATCCCTCAG  Reverse:AGGATGGAAGGCATGTATGTTTG | NM_021911.3 | 83 bp | 59 | Exon 4-5 |
| GAP43 | Probe:CCAGCCTCCAACGGAGACT  Forward:GCCGCAGAGGATGCTGCT  Reverse:CTATGTTCTCTTCAGCTTGGCTGC | NM_002045.4 | 82 bp | 60 | Exon 2-3 |
| GAPDH | Probe:AGGCTGGGGCTCAT  Forward:GGAGTCCACTGGCGTCTTCA  Reverse:GGGCAGAGATGATGACCCTTT | NM_002046.7 | 83 bp | 59 | Exon 3-4 |
