# Supplementary material for: miRNA-associated gene networks reveal potential candidate markers for Alzheimer’s disease
Source: Front Mol Biosci. 2026 Mar 6;12:1699404. doi: 10.3389/fmolb.2025.1699404 (PMC13002409; doi:10.3389/fmolb.2025.1699404)
Supplement: Supplementary file 1 [file Supplementaryfile1.zip › Supplementary Tables/Supplementary Table 10.doc]

**Supplementary Table 10. Hub Gene Expression by qRT-PCR.**

| Demographics | AD (n=85) | Controls (n=74) | *t* | *p* | *q* |
| --- | --- | --- | --- | --- | --- |
| BDNF mRNA (2^–ΔΔCT^) | 1.19±0.48 | 1.08±0.51 | 1.29 | 0.2 | 0.333 |
| SLC32A1 mRNA(2^–ΔΔCT^) | 1.47±0.72 | 1.12±0.53 | 3.25 | 0.001** | 0.005 |
| CALB1 mRNA(2^–ΔΔCT^) | 1.12±1.11 | 1.25±0.98 | -0.73 | 0.467 | 0.612 |
| GAD1 mRNA(2^–ΔΔCT^) | 1.40±0.56 | 1.09±0.45 | 3.83 | <0.001** | 0.005 |
| GAD2 mRNA(2^–ΔΔCT^) | 1.24±0.96 | 1.20±0.75 | 0.26 | 0.8 | 0.800 |
| GAP43 mRNA(2^–ΔΔCT^) | 0.91±0.49 | 1.05±0.53 | -1.62 | 0.11 | 0.244 |
| SYNPR mRNA(2^–ΔΔCT^) | 1.13±0.43 | 1.08±0.43 | 0.68 | 0.49 | 0.612 |
| GABRA2 mRNA(2^–ΔΔCT^) | 1.04±0.59 | 1.14±0.67 | -0.97 | 0.33 | 0.471 |
| GABRA1 mRNA(2^–ΔΔCT^) | 1.17±0.73 | 1.13±0.54 | 0.32 | 0.75 | 0.800 |
| SNAP25 mRNA(2^–ΔΔCT^) | 1.19±0.65 | 1.08±0.41 | 1.3 | 0.2 | 0.333 |

BDNF: Brain-Derived Neurotrophic Factor; SLC32A1: solute carrier family 32 member 1; CALB1:calbindin 1; GAD1:glutamate decarboxylase 1; GAD2: glutamate decarboxylase 2; GAP43: growth associated protein 43; SYNPR: synaptoporin; GABRA1：gamma-aminobutyric acid type A receptor subunit alpha1; GABRA2：gamma-aminobutyric acid type A receptor subunit alpha 2; SNAP25: synaptosome associated protein 25.
